# Optimal Timing of Anterior Cruciate Ligament Reconstruction in Patients With Anterior Cruciate Ligament Tear: A Systematic Review and Meta-analysis

**DOI:** 10.1001/jamanetworkopen.2022.42742

**Published:** 2022-11-17

**Authors:** Xianyue Shen, Tong Liu, Shenghao Xu, Bo Chen, Xiongfeng Tang, Jianlin Xiao, Yanguo Qin

**Affiliations:** 1 Department of Orthopedics, The Second Hospital of Jilin University, Changchun, Jilin Province, China; 2 Department of Orthopedics, China-Japan Union Hospital of Jilin University, Changchun, Jilin Province, China

## Abstract

**Importance:**

The timing of surgery has been regarded as a key factor in anterior cruciate ligament reconstruction (ACLR), and early vs delayed ACLR remains a controversial topic.

**Objective:**

To synthesize up-to-date published data from randomized clinical trials (RCTs) comparing early vs elective delayed ACLR for patients with ACL deficiency, in terms of clinical outcomes and complications.

**Data Sources:**

The PubMed, Cochrane Library, and Web of Science databases were systematically searched until September 9, 2022.

**Study Selection:**

All published RCTs comparing clinical and functional outcomes and complications associated with early ACLR vs elective delayed ACLR.

**Data Extraction and Synthesis:**

Two reviewers independently extracted relevant data and assessed the methodological quality following the PRISMA guidelines.

**Main Outcomes and Measures:**

Due to the clinical heterogeneity, the random-effects model was preferred. The primary outcomes were functional outcomes and complications. The Mantel-Haenszel test was used to evaluate dichotomous variables and the inverse variance method was used to assess continuous variables.

**Results:**

This meta-analysis included 972 participants in 11 RCTs stratified by follow-up duration. The following factors did not differ between early and delayed ACLR: operative time (mean difference, 4.97; 95% CI, −0.68 to 10.61; *P* = .08), retear (OR, 1.52; 95% CI, 0.52-4.43; *P* = .44), and infection (OR, 3.80; 95% CI, 0.77-18.79; *P* = .10). There were also no differences between groups in range of motion, knee laxity, International Knee Documentation Committee (IKDC rating scale), and Tegner score. IKDC score (mean difference, 2.77; 95% CI, 1.89-3.66; *P* < .001), and Lysholm score at 2-year follow-up (mean difference, 2.61; 95% CI, 0.74-4.48; *P* = .006) significantly differed between early and delayed ACLR. In addition, the timing of surgery was redefined in the included RCTs and subgroup analyses were performed, which validated the robustness of the principal results.

**Conclusion and Relevance:**

This systematic review and meta-analysis found that early ACLR was not superior to delayed ACLR in terms of most factors analyzed, except for IKDC and Lysholm scores. This information should be available to patients with ACL deficiency and clinicians as part of the shared decision-making process of treatment selection.

## Key Points

**Question:**

Is the timing of anterior cruciate ligament reconstruction (ACLR) associated with clinical outcomes in patients with ACL tears?

**Findings:**

This systematic review and meta-analysis of 11 randomized clinical trials involving 972 participants found that, compared with elective delayed ACLR, early ACLR was not associated with improved functional outcomes nor fewer complications, regardless of the follow-up duration.

**Meaning:**

These findings suggest that the timing of ACLR surgery may not influence functional outcomes and postoperative complications; this information should be available to patients with ACL deficiency and their surgeons as part of the shared decision-making process of treatment selection.

## Teaser

This systematic review and meta-analysis examines randomized clinical trials comparing early vs elective delayed anterior cruciate ligament (ACL) reconstruction for patients with ACL deficiency in terms of clinical outcomes and complications.

## Introduction

Anterior cruciate ligament reconstruction (ACLR) is a common surgical procedure that can effectively treat ACL injuries; approximately 200 000 ACLR procedures are performed annually in the United States.^1,2^ It has been suggested that timing of ACLR surgery is a key factor determining clinical outcomes and postoperative complications.^3,4,5,6^

The effects of the timing of ACLR surgery on postoperative knee function and clinical outcomes continue to be heavily debated. Early ACLR may reduce postoperative complications in patients with ACL deficiency,^7,8^ whereas elective delayed reconstruction can decrease the risk of knee fibrosis and improve clinical results.^9,10^ However, delayed ACLR may be associated with muscle atrophy and reduced strength, which may prevent early rehabilitation.^11^ Some proponents of early ACLR believe that early intervention is beneficial for patients with ACL deficiency.^12^

Although some meta-analyses on the optimal timing of ACLR have been conducted,^13,14,15^ these analyses included low-level studies, with heterogeneous follow-up durations and no long-term results. Recently, high-quality research studies^16,17^have provided new evidence on this topic. In 2021, Reijman et al^17^ assessed 165 participants with ACL deficiency, and reported that compared with delayed reconstruction, early ACLR was associated with improved knee function and movement ability at the 2-year follow-up assessment. Nevertheless, no consensus has been reached on whether early or delayed ACLR should be used for the treatment of ACL injuries. Therefore, the present systematic review and meta-analysis aimed to synthesize the latest research comparing the outcomes of early and elective delayed ACLR, so as to help orthopedists and patients make evidence-based decisions on the timing of ACLR.

## Methods

### Search Strategy and Trial Selection

This meta-analysis and systematic review followed the Preferred Reporting Items for Systematic Reviews and Meta-analyses (PRISMA) reporting guideline.^18^ The search strategy and inclusion and exclusion criteria are reported in the eMethods in Supplement 1. This study was exempted from ethical review by the of the Second Hospital of Jilin University institutional review board because it was a secondary synthesis of published data.

### Data Extraction, Synthesis, and Measurement of Outcomes

Two reviewers (X.Y.S. and S.H.X.) independently extracted the relevant data from the included trials and imported the extracted data into a spreadsheet (Excel 2019, Microsoft), which was reviewed by another senior reviewer (T.L.). Details of the data are reported in the eMethods in Supplement 1.

### Study Quality Assessment and Risk-of-Bias Assessment

The methodological quality of the RCTs was assessed using the Cochrane Collaboration tool for assessing risk of bias with 7 standard criteria. Two reviewers (X.F.T. and B.C.) independently evaluated the risk of bias of the included trials, and any disagreements were discussed until a consensus was reached. The risk-of-bias assessments are reported in eTable 2 in Supplement 1. Publication bias could not be assessed using funnel plots, as none of the meta-analyses included more than 10 studies.

### Statistical Analysis

Meta-analysis of the extracted data was performed using Review Manager version 5.3 (The Cochrane Collaboration) from January to September 2022. The Mantel-Haenszel test was used to evaluate dichotomous variables, which were expressed as odds ratios (ORs), and the inverse variance method was used to assess continuous variables, which were expressed as mean differences. The 95% CI was calculated for each effect size. Two-sided *P* < .05 was considered statistically significant. Details of the statistical analyses are reported in the eMethods in Supplement 1.

## Results

### Search Results and Studies Included

**Figure 1.**
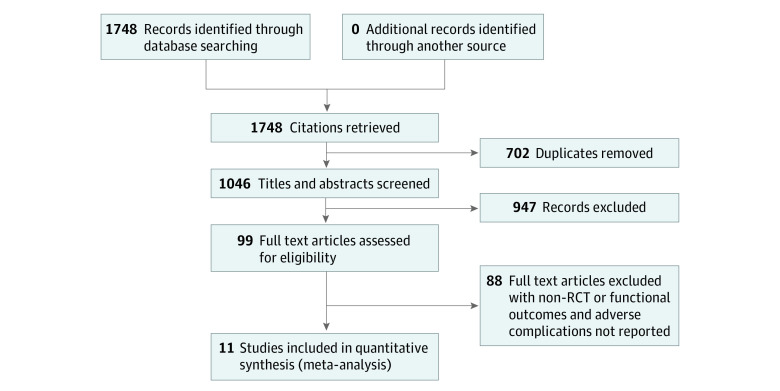
Flowchart of the Selection Process for the Included Randomized Clinical Trials

The PRISMA flowchart of the article-selection process is presented in Figure 1. The electronic search yielded 1748 potentially relevant studies. After the removal of duplicates, 702 studies were screened and reviewed by their titles and abstracts. Then the full texts of the remaining 99 studies were evaluated for eligibility based on the inclusion and exclusion criteria. Finally, 11 RCTs^16,17,19,20,21,22,23,24,25,26,27^ were included in the quantitative data synthesis.

### Study, Participant, and Intervention Characteristics

The participant, intervention, and study characteristics of the 11 included RCTs are provided in the Table. The total number of participants in the included RCTs was 972 (early ACLR: 487; delayed ACLR: 379; only rehabilitation: 106), and the follow-up durations ranged from 0.5 to 5 years after ACLR. All included RCTs reported the preoperative rehabilitation principles of elective delayed ACLR with the goal of achieving full range of motion and muscle strength. Different broad time cutoffs were used to define early and delayed reconstruction in the included RCTs. The definition of early ACLR ranged from 8 days to 10 weeks, while that of delayed ACLR ranged from 4 weeks to greater than 3 months. Moreover, there was an overlap of these time intervals between some studies.

**Table.** Characteristics of RCTs Included in the Analysis

Source	Study design	Randomization	Definition of surgery time		Follow-up	Inclusion criteria	Preoperative rehabilitation principle of elective delay ACLR	Postoperative rehabilitation principle
			Early	Elective delay				
Meighan et al,^19^ 2003	RCT	Envelope	<2 wk	8-12 wk	52 wk	Acute ACL tear; age <35 y; no MCL injury; no previous ligament injury; no previous meniscal tear	Preoperative physiotherapy to restore knee ROM	Identical postoperative program of accelerated rehabilitation
Bottoni et al,^20^ 2008	RCT	Sealed envelopes	Within 21 d	>6 wk	366 d (185-869)	Age ≥18 y; acute ACL tear; no prior ligamentous surgery on the index knee or a concomitant posterior cruciate, fibular collateral, or posterolateral corner ligamentous knee injury	A supervised rehabilitation protocol that emphasized quadriceps muscle strengthening and restoration of a full ROM; return to full active duty or recreational sports were not permitted; concomitant MCL injury, a hinged knee brace was prescribed that allowed sagittal plane motion but limited valgus motion	Patients with meniscal repair or microfracture, partial weight-bearing with crutches for 4 wk. The drop-lock brace applied in the operating room was used continuously for 4 to 6 wk until good quadriceps control was restored. Subsequently, no functional bracing was used for any patient. Return to sports at 4 to 6 mo
Raviraj et al,^21^ 2010	RCT	Computer-generated randomization sequence	<2 wk	4-6 wk	32 mo (26-36)	MRI-confirmed ACL injury; age >18 y; no bilateral injury, long-bone fracture, additional procedures, previous surgery, or Outerbridge grade III/IV	ROM exercises and hamstrings and quadriceps strengthening exercises; a hinged brace was applied which was locked in extension for walking, with weight-bearing allowed as tolerated; when not walking patients were allowed a full ROM in the brace	Patients were placed in a hinged knee brace and touch weight-bearing was allowed for 2 wk, with the ROM was restricted to 0-90° of flexion; subsequently, weight-bearing as tolerated with the ROM no longer restricted, but with wearing of the brace for 6 wk
Frobell et al,^22^ 2010	RCT	Sealed envelopes	Within 10 wk		2 y	Age 18-35 y; <4 wk knee of ACL tear; Tegner 5-9; no LCL, MCL, or full-thickness cartilage injury; no meniscal tear (grade III); no previous knee surgery; no bilateral injury; no PL complex injury; no pregnancy; no DVT or coagulative problems; no claustrophobia; no steroid use, systemic disease, or unstable meniscal lesion	Structured rehabilitation	Structured rehabilitation
Frobell et al,^23^ 2013	RCT	Sealed envelopes	Within 10 wk		5 y	Age 18-35 y; <4 wk knee of ACL tear; Tegner 5-9; no LCL, MCL, or full-thickness cartilage injury; no meniscal tear (grade III); no previous knee surgery; no bilateral injury; no PL complex injury; no pregnancy; no DVT or coagulative problems; no claustrophobia; no steroid use, systemic disease, or unstable meniscal lesion	Structured rehabilitation	Structured rehabilitation
Chen et al,^24^ 2015	RCT	Rolling a dice	3-7 wk	6-11 mo	5 y	Unilateral primary ACL rupture; age <50 y; no untreated ligament injury, bilateral injury, previous surgery, or infection	Cryotherapy for local swelling in 2 d after injury; hot compress (starting 2 d after injury and continuing up to 2 wk until the swelling or pain recedes); CPM knee joint exercise once a day; actively contract the quadriceps and peroneal muscle 100 times/d; wear an adjustable knee brace to support partial weight bearing	1 Day after surgery, initial exercises, with ROM 0–30°. After 3 d to 1 wk, isometric and isotonic quadriceps exercises; after 1 wk, partial weight-bearing activities assisted by crutches; in the first month, achieve full weight-bearing activities without crutches, and to begin balance and gait training assisted by a walker; at 2-3 mo, locomotor activity without any auxiliary tools; after 1 y or more, recreational or non-competitive sports
Manandhar et al,^25^ 2018	RCT	On the basis of odd and even hospital numbers	Within 3 wk	>6 wk	6 mo	ACL insufficiency with or without associated chondral (Outerbridge I & II) injuries, meniscal injuries	A physiotherapy program where the quadriceps was strengthened and the ROM restored to 120° of flexion	Next day, mobilization and partial weight bearing with a pair of crutches, ROM set 0-90°of flexion for the first 3 wk; partial weight bearing with a pair of crutches for 2 wk; recumbent cycling was started at 3 wk; half squats, slow walking and jogging was allowed; after 3 mo, swimming and side running progressing to zig zag running, single leg presses and single half squats. At the 4th month, balance training using wobble board. At the 5th month, plyometrics and skill exercises. After 6 mo, start playing
Eriksson et al,^26^ 2018	RCT	Sealed envelope	Within 8 d	6-10 wk	6 mo	Unilateral primary ACL injury; age 18-40 y; no previous knee injury; Tegner >6; no additional meniscus or cartilage damage; no LCL injury; no MCL injury; no PCL insufficiency and no signs of OA	Preoperative physiotherapy to restore normal ROM and to preserve muscle strength	Day 1, full weight-bearing. A brace for patients with suturing of menisci. The brace had a fixed ROM 0-60° for 4 wk and 0-90° for another 2 wk, full weight-bearing with the support of crutches during the first 3 wk. Closed-chain exercises and ROM training was initiated within 1 wk of surgery. Open-chain exercises after 6 wk, running allowed after 14 wk and resumption of sport activity after Biodex® testing showed 90% strength in injured leg compared to the contralateral leg, but never earlier than 6 mo
von Essen et al,^16^ 2020	RCT	Sealed envelope technique	Within 8 d	6-10 wk	24 mo	Acute ACL injury; age 18-40 y; Tegner >6; no major cartilage or meniscus injury; no signs of OA; no MCL injury or multiple ligament injuries	Preoperative physiotherapy to restore normal ROM and to preserve muscle strength	Closed-chain exercises and ROM training was initiated within 1 wk after the surgery. Open chain exercises were allowed after 6 wk, running allowed after 14 wk and resumption of sport activity after Biodex® testing showed 90% strength in injured leg compared to the contralateral leg, but never earlier than 6 mo
von Essen et al,^27^ 2020	RCT	Sealed envelope technique	Within 8 d	6-10 wk	12 mo	Acute ACL injury; age 18-40 y; Tegner >6; no major cartilage or meniscus injury; no signs of OA; no MCL injury or multiple ligament injuries	Preoperative physiotherapy to restore normal ROM and to preserve muscle strength	Closed-chain exercises and ROM training was initiated within 1 wk after the surgery. Open chain exercises were allowed after 6 wk, running allowed after 14 wk and resumption of sport activity after Biodex® testing showed 90% strength in injured leg compared to the contralateral leg, but never earlier than 6 mo
Reijman et al,^17^ 2021	RCT	Computer generated randomization lists	Within 6 wk	>3 mo	24 mo	Primary ACL rupture; age 18-65 y; no history of ACL rupture of the contralateral knee; no other disorder affecting the activity of the lower limb; no dislocated bucket handle lesion of the meniscus with an extension deficit, or insufficient command of the Dutch language	A supervised physical therapy program	According to the recommendations of the Dutch ACL guideline, patients were referred for physical therapy until good functional control was achieved

Abbreviations: ACL, anterior cruciate ligament; DVT, deep venous thrombosis; LCL, lateral collateral ligament; MCL, medial collateral ligament; OA, osteoarthritis; RCT, randomized clinical trial; ROM, range of motion.

### Graft Selection

Among all the included RCTs, in both the early and delayed ACLR groups, the most commonly used ACL grafts were hamstring grafts (772 of 862 grafts [83.3%]) and bone-patellar tendon-bone grafts (85 of 862 grafts [9.8%]). Only 1 study^24^ used a ligament augmentation and reconstruction system as an alternative ACL graft during the reconstruction surgery, accounting for 55 of 862 grafts (6.4%).

### Mechanisms of Injury and Associated Lesions

Sports-related injuries and traffic accidents were the most common causes of ACL tears in the included studies (eTable 3 in Supplement 1). Of the 11 studies, 9 studies^16,17,19,20,21,22,25,26,27^ reported that the ACL injury was accompanied by a meniscus injury (incident rate: 54.2% [432 of 797]) and 7 studies^16,17,20,21,25,26,27^ reported that the ACL injury was accompanied by a cartilage injury (incidence rate: 29.9% [193 of 645]).

### Domain 1: Hospital Admission Impact

#### Operative Time

Of the 11 included studies, 6 studies^16,19,20,21,26,27^ reported operative times of early and elective delayed ACLR. SD data were lacking in 1 study,^19^ so it was not included in the meta-analysis. Meta-analysis of the other 4 studies showed no statistically significant difference in operative time between 2 groups (mean difference, 4.97; 95% CI, −0.68 to 10.61; *P* = .08) (eFigure 1 in Supplement 1).

### Domain 2: Objective Functional Outcomes

#### Range of Motion

Data on range of motion from 4 studies^16,20,26,27^ were included in the meta-analysis. Results of subgroup analyses stratified by follow-up duration showed no significant difference in knee extension and flexion deficit between 2 groups (eFigure 2 in Supplement 1).

#### Knee Laxity

**Figure 2.**
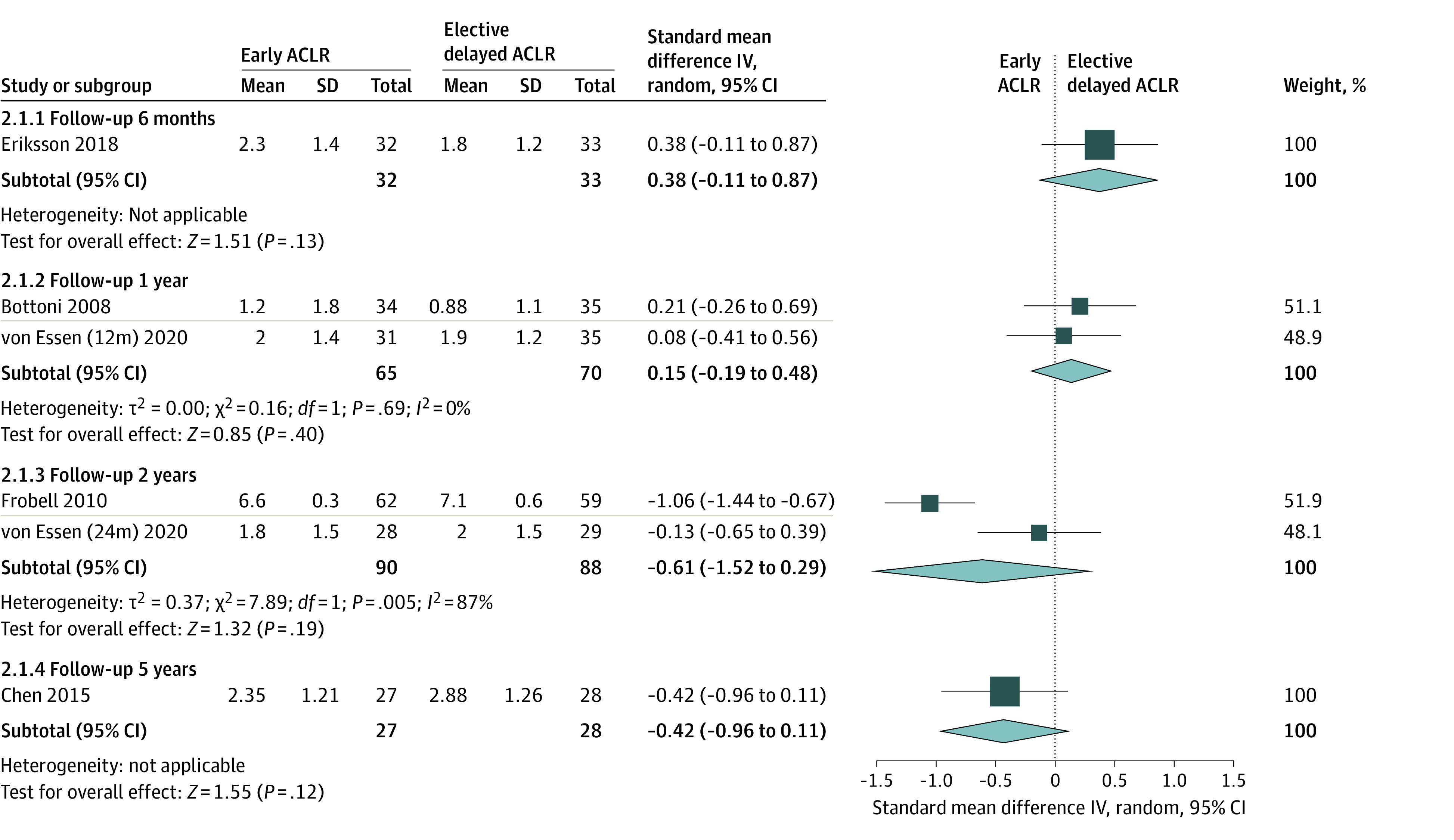
Forest Plot Depicting Knee Laxity After Early ACLR vs Elective Delayed ACLR ACLR indicates anterior cruciate ligament reconstruction; IV, inverse variance.

Seven studies reported data on knee laxity after ACLR, which was measured using a KT-1000 arthrometer in 4 studies^20,21,22,24^ and a Rollimeter in 3 studies.^16,26,27^ At each follow-up time point, the results of subgroup analyses showed no significant difference in knee laxity between 2 groups (Figure 2).

### Domain 3: Subjective Functional Outcomes

#### Lysholm Score

**Figure 3.**
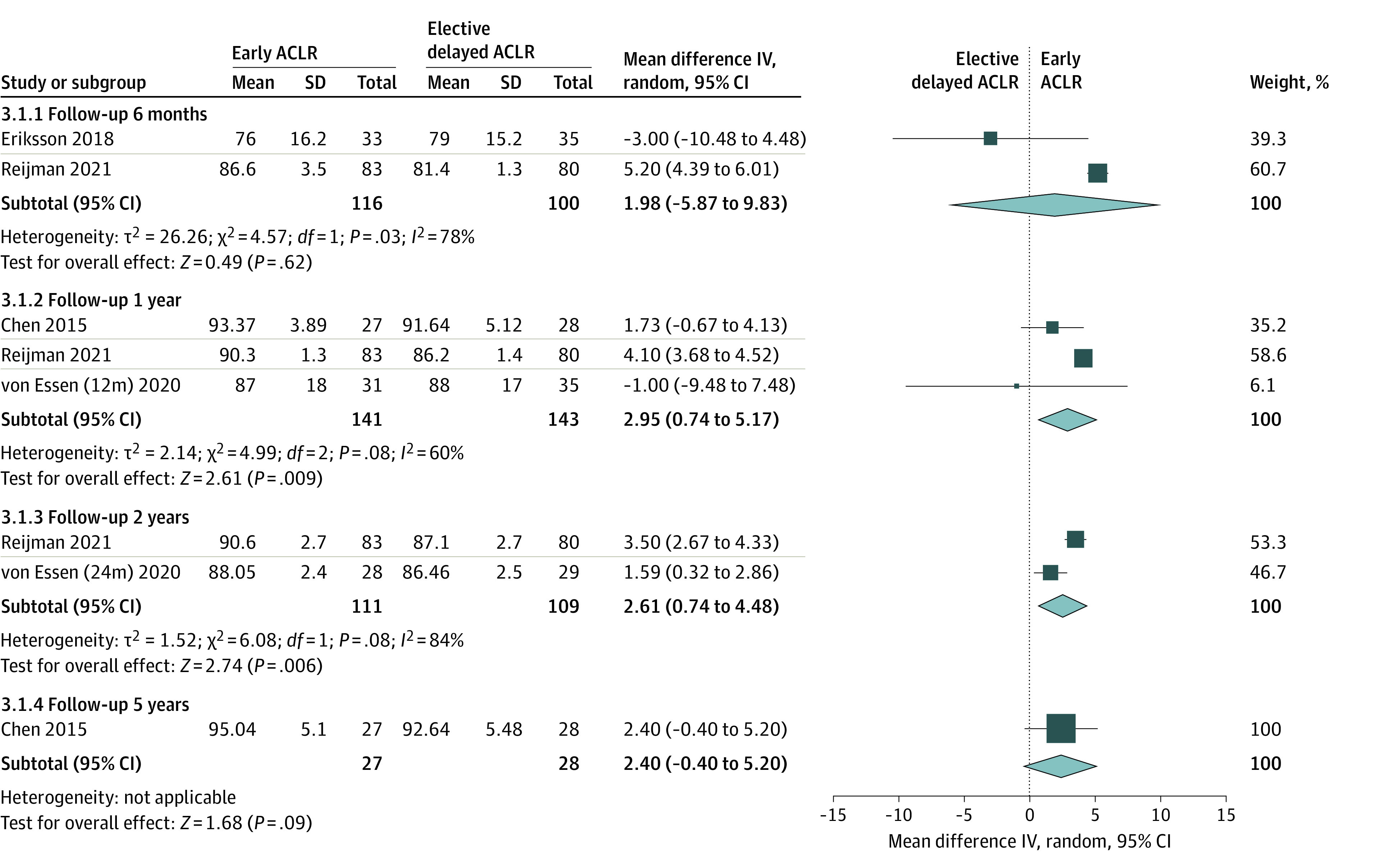
Forest Plot Depicting Lysholm Score After Early ACLR vs Elective Delayed ACLR ACLR, anterior cruciate ligament reconstruction; IV, inverse variance.

Seven studies^16,17,20,21,24,26,27^ used Lysholm score to evaluate knee function after ACLR. In one study,^21^ the authors reported that Lysholm score of the knee at 32 months after surgery did not significantly differ between early and delayed ACLR. Two studies^20,21^ that reported Lysholm scores as means and ranges could not be included in the meta-analysis. After the included studies were stratified by follow-up duration, the pooled results showed no significant difference in Lysholm score between 2 groups at 6-month and 5-year follow-up evaluations; however, Lysholm scores were significantly higher in the early group than the delayed ACLR group at 1-year follow-up (mean difference, 2.95; 95% CI, 0.74-5.17; *P* = .009) and 2-year follow-up (mean difference, 2.61; 95% CI, 0.74-4.48; *P* = .006) assessments (Figure 3).

#### Tegner Score

Of the 11 included studies, 10 studies^16,19,20,21,22,23,24,25,26,27^ reported Tegner score of the knee after early and elective delayed ACLR, but only 2 studies^24,25^ were included in the meta-analysis. The pooled results suggested that Tegner score did not significantly differ between study groups, after stratification by follow-up duration (eFigure 3 in Supplement 1).

#### IKDC Score or IKDC Rating Scale

In 2 studies^17,25^ with 6-month follow-up, meta-analysis showed that early ACLR was associated with higher International Knee Documentation Committee (IKDC) scores (mean difference, 2.77; 95% CI, 1.89-3.66; *P* < .001) (eFigure 4 in Supplement 1). In the remaining 5 studies,^16,19,24,26,27^ the postoperative knee function was assessed using IKDC rating scale. The pooled results showed that the proportion of patients with a normal grade did not significantly differ between 2 groups (eFigure 4 in Supplement 1).

#### KOOS Subscales

Of the 11 studies, 6 studies^16,17,22,23,26,27^ used Knee Injury and Osteoarthritis Outcome Score (KOOS) subscales to assess postoperative knee function. Because these studies did not express these data in a consistent way, we could not perform a meta-analysis. However, in studies with follow-up durations of 0.5 to 5 years, no significant difference in KOOS subscales was found between 2 groups. eFigure 5 in Supplement 1 shows the KOOS subscales across 4 studies,^16,17,22,23^ and shows that the KOOS score was comparable between 2 groups.

### Domain 4: Major Adverse Events

#### Retear and Infection

A total of 10 RCTs^16,17,19,20,21,22,23,24,25,26^ reported the occurrence of adverse events (eTable 4 in Supplement 1). The most frequently reported complications were ACL retear and infection. The pooled results^16,17,19,20,22,23^ showed that the probabilities of ACL retear after early ACLR was 3.9% and after delayed ACLR was 2.6% with no significant difference (OR = 1.52; 95% CI, 0.52-4.43; *P* = .44) (eFigure 6 in Supplement 1). The pooled results^19,20,21,24,25^ showed that the incidence of infection was 2.8% after early ACLR 0% after delayed ACLR, but it did not reach statistical significance (OR = 3.80; 95% CI, 0.77-18.79; *P* = .10) (eFigure 7 in Supplement 1).

### Subgroup Analyses

**Figure 4.**
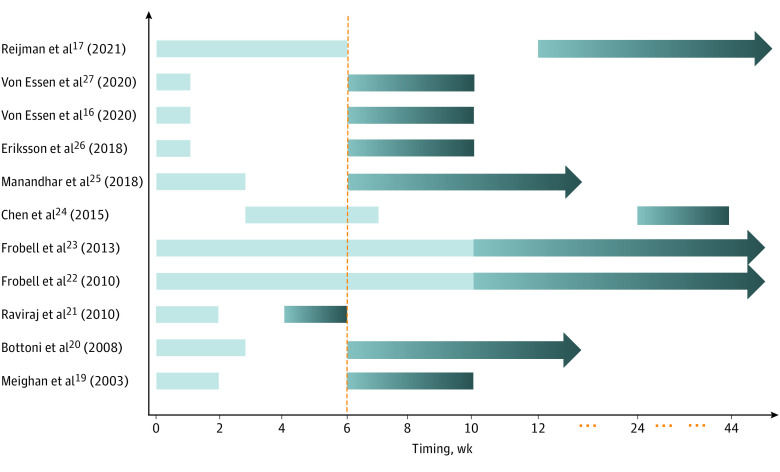
Detailed Timings of Surgery in the Included Randomized Clinical Trials and Redefinition of the Surgical Timings The vertical dashed line represents the redefined cutoff values of anterior cruciate ligament reconstruction operation timing: early was defined as within 6 weeks and delayed was defined as after 6 weeks.

The overlap in the time cutoffs used to define early and delayed reconstruction between some studies led to high heterogeneity. To minimize this overlap, we excluded 4 studies^21,22,23,24^ and redefined early ACLR as an injury-to-surgery time of within 6 weeks and delayed ACLR as greater than 6 weeks (Figure 4). The overall results of the subgroup analyses showed that at each follow-up time point, range of motion, knee laxity, IKDC rating scale, Tegner score, and adverse complications of the affected knee did not significantly differ between 2 groups. In contrast, significant differences were found between 2 groups in terms of the Lysholm score at 2-year follow-up (mean difference, 2.61; 95% CI, 0.74-4.48; *P* = .006) and the IKDC score (*P* < .001; (eFigure 8, 9, 10, 11, 12, 13, and 14 in Supplement 1).

## Discussion

### Principal Findings

The current meta-analysis and systematic review found that there were no significant differences in operative time, range of motion, knee stability, Tegner score, IKDC rating scale, and incidence of the most frequent complications between early and elective delayed ACLR, regardless of the follow-up duration. Early ACLR was superior to elective delayed ACLR in terms of the Lysholm score at 2 years and the IKDC score. These results were validated using subgroup analyses. This information may help orthopedic surgeons and patients with ACL deficiency to make an informed choice between early and delayed ACLR. Our findings may be used to reduce the anxiety of patients awaiting surgery for ACL tears, because the differences in functional outcomes and adverse events between the 2 approaches are not obvious.

### Timing of ACLR

It is generally accepted that the timing of ACLR is an important factor determining postoperative functional outcomes.^28,29^ Although many studies have investigated the effect of the timing of surgical reconstruction on patients’ functional outcomes, the optimal timing remains controversial. Currently, there exist no unified definitions of early vs delayed surgery, and various studies use their own time cutoffs to define early and delayed reconstruction. For example, in the RCT by Barenius et al,^30^ early reconstruction was defined as an injury-to-surgery time of less than 5 months, and reconstruction beyond 5 months was defined as delayed reconstruction. In the prospective clinical trial by Fithian et al,^12^ early ACLR was defined as surgery within 3 months. Among the studies in our meta-analysis, early ACLR was variously defined as an injury-to-surgery time ranging from 8 days to 10 weeks. Such a large difference would lead to considerable heterogeneity in the study conclusions. Therefore, we redefined early ACLR as an injury-to-surgery time of within 6 weeks to minimize the overlap among the different definitions and make our conclusion more standardized and credible. This definition is also consistent with the definitions used in some relevant reports.^17,31^ A uniform definition of early ACLR should be established in the future, as this would help reduce the apparent heterogeneity in reporting.

### Comparison With Other Studies

Although many studies have investigated the notably improved knee functional outcomes of ACLR in patients with ACL deficiency, the effect of the timing of ACLR on functional improvement is unclear. To explore the effects of the timing of ACLR on knee motion and stability, Hunter et al^32^ divided 185 patients into 4 subgroups based on the injury-to-surgery interval and concluded that no significant differences in extension and flexion were present among the subgroups at any time.

However, few studies have attempted to determine the optimal timing of ACLR. Most studies have focused on comparing the effects of early and late surgery. Some studies conducted before the 21st century^9,33,34^ have reported that patients with ACL injury could obtain better joint stability and less movement limitation after delayed rather than early ACLR. Paradoxically, other studies have suggested that early ACLR is associated with better clinical outcomes.^26,35^ The aforementioned differences may be attributed to differences in rehabilitation protocols. Effective modern early rehabilitation after ACLR plays an important role in functional outcomes. Furthermore, we speculate that preoperative physiotherapy in the delayed ACLR group would also benefit the clinical outcomes.

Some reviews have relied on scarce or low-quality evidence in an attempt to aggregate the existing studies and analyze the timing of ACLR. For example, Smith et al^13^ included 6 studies in a pooled analysis and concluded that no significant differences in outcomes were present between the early and delayed ACLR. Although this conclusion is consistent with our findings, only 2 of the 6 included studies were RCTs, and the literature had substantial methodological limitations. In contrast, the studies we included were all high-level RCTs with strict inclusion and exclusion criteria, and superior levels of evidence. Moreover, we redefined the timing of ACLR and conducted subgroup analyses, which solved the problem of overlapping time intervals and validated the robustness of our conclusions.

In a recent meta-analysis involving eight RCTs, Deabate et al^36^ found that early ACLR provides similarly good functional outcomes as delayed ACLR, without increasing the risk of complications such as arthrofibrosis and range of motion limitation. However, the follow-up durations in the included studies were heterogeneous, and long-term outcomes were lacking. To reduce heterogeneity, we stratified the results of the included studies by follow-up duration. We found no differences between early and elective delayed ACLR in terms of range of motion, knee stability, Tegner score, IKDC rating scale, and complications. We also found that early ACLR was superior to elective delayed ACLR in terms of the Lysholm score at 2 years and the IKDC score, which differs from the results of Smith et al^13^ and Deabate et al.^36^ These differences may be attributed to the inclusion of different items of interest in different scoring systems.

Surprisingly, the results of this meta-analysis differ from those reported by Ramski et al,^37^ who favored early operative treatment for pediatric patients with ACL tears over delayed or nonoperative treatment. The authors reported that early ACLR yields better knee stability, fewer meniscus tears, and higher rates of return to previous activity levels than nonoperative treatment and delayed ACLR. The discrepancy may be attributed to differences in the skeletal maturity of the participants. In addition, many outcome measurements were developed for adult patients, and the evaluation of these indicators in pediatric patients may be less accurate. Thus, the optimal timing of ACLR may differ among patients with ACL deficiency with different levels of bone maturity. Future studies should evaluate the effect of operation timing in patients with ACL deficiency and different bone maturity.

The current study found that there was no significant difference between early and elective delayed ACLR with respect to ACL retear and infection and the rates of these 2 complications in the 2 study groups were largely consistent with those reported in the literature.^38,39,40,41,42,43^ This suggests that the timing of surgery has little effect on the postoperative complications of ACLR.

### Strengths and Limitations

Our work has many strengths. To our knowledge, the present meta-analysis and systematic review has pooled the largest-to-date RCT data set to evaluate the effect of the timing of ACLR on knee functional outcomes. Our study is the first to meticulously analyze knee functional outcomes at different follow-up time points to capture the effects of early and delayed ACLR in a relatively timely and dynamic manner. We followed strict inclusion and exclusion criteria to minimize confounding factors in the RCT population.^44^ In addition, we redefined the timing of surgery in the included RCTs and performed additional subgroup analyses to validate the robustness of our principal findings.

This study also has some limitations. First, participant characteristics and technical details of the reconstruction could not be pooled in meta-analyses; ideally, these factors should be analyzed individually.^45^ Second, although the latest RCTs were included, some of the plotted results were reported in only a few trials, and some required estimates of SD; the quality of the evidence remained low. Third, a certain degree of heterogeneity in preoperative strategies, postoperative rehabilitation, and physiotherapy management was present among the RCTs, although all modern accelerated rehabilitation programs appear to be effective. Fourth, our study did not explore the effect of graft selection and associated lesions on the clinical outcomes of ACLR. This is because most of the included RCTs used autografts, making subgroup analyses stratified by graft selection difficult. Furthermore, among the included RCTs, the associated lesions were mixed, due to which their potential effects on postoperative functional outcomes could not be analyzed. Finally, we analyzed some relevant data reported in the same RCT at different follow-up time points, which may lead to reuse of the same data set, but the effect of this limitation is uncertain.

## Conclusions

The present meta-analysis and systematic review of RCTs showed that there were no significant differences in operative time, range of motion, knee stability, Tegner score, IKDC rating scale, and adverse complications, at any time, between early and elective delayed ACLR. This information should be available to patients with ACL deficiency and their clinicians as part of the shared decision-making process of treatment selection.

## Notes

Supplement 1.
eMethods
**eTable 1.** Study Inclusion and Exclusion Criteria **eTable 2.** Risk of Bias of Assessment for the Included RCTs Using Cochrane Collaboration’s Tool **eTable 3.** Patient and Treatment Characteristics of Included Trials **eTable 4.** Summary of Adverse Events After Early and Delayed ACLR in Included RCTs **eFigure 1.** Forest Plot Depicting the Operative Time of Early ACLR Versus Elective Delayed ACLR **eFigure 2.** Forest Plots Depicting the Extension Deficit and Flexion Deficit of Early ACLR Versus Elective Delayed ACLR **eFigure 3.** Forest Plot Depicting the Tegner Score of Early ACLR Versus Elective Delayed ACLR **eFigure 4.** Forest Plots Depicting the IKDC Score and IKDC Rating Scale of Early ACLR Versus Elective Delayed ACLR **eFigure 5.** KOOS Subscales for Early ACLR and Elective Delayed ACLR Cohorts From Four Included Studies **eFigure 6.** Forest Plot of the Results of Re-Tear of Early ACLR Versus Elective Delayed ACLR **eFigure 7.** Forest Plot of the Results Infection of Early ACLR Versus Elective Delayed ACLR **eFigure 8.** Forest Plots Depicting the Extension Deficit and Flexion Deficit of Early ACLR Versus Elective Delayed ACLR After Redefinition **eFigure 9.** Forest Plot Depicting the Knee Laxity of Early ACLR Versus Elective Delayed ACLR After Redefinition **eFigure 10.** Forest Plot Depicting the Lysholm Score of Early ACLR Versus Elective Delayed ACL After Redefinition **eFigure 11.** Forest Plot Depicting the Tegner Score of Early ACLR Versus Elective Delayed ACLR After Redefinition **eFigure 12.** Forest Plots Depicting the IKDC Score and IKDC Rating Scale of Early ACLR Versus Elective Delayed ACLR After Redefinition **eFigure 13.** Forest Plot of the Results of Re-Tear of Early ACLR Versus Elective Delayed ACLR After Redefinition **eFigure 14.** Forest Plot of the Results Infection of Early ACLR Versus Elective Delayed ACLR After Redefinition

Supplement 2. Data Sharing Statement
