# Supplementary material for: Optimal Timing of Anterior Cruciate Ligament Reconstruction in Patients With Anterior Cruciate Ligament Tear: A Systematic Review and Meta-analysis
Source: JAMA Netw Open. 2022 Nov 17;5(11):e2242742. doi: 10.1001/jamanetworkopen.2022.42742 (PMC9672975; doi:10.1001/jamanetworkopen.2022.42742)
Supplement: Supplement 1. — eMethods eTable 1. Study Inclusion and Exclusion Criteria eTable 2. Risk of Bias of Assessment for the Included RCTs Using Cochrane Collaboration’s Tool eTable 3. Patient and Treatment Characteristics of Included Trials eTable 4. Summary of Adverse Events After Early and Delayed ACLR in Included RCTs eFigure 1. Forest Plot Depicting the Operative Time of Early ACLR Versus Elective Delayed ACLR eFigure 2. Forest Plots Depicting the Extension Deficit and Flexion Deficit of Early ACLR Versus Elective Delayed ACLR eFigure 3. Forest Plot Depicting the Tegner Score of Early ACLR Versus Elective Delayed ACLR eFigure 4. Forest Plots Depicting the IKDC Score and IKDC Rating Scale of Early ACLR Versus Elective Delayed ACLR eFigure 5. KOOS Subscales for Early ACLR and Elective Delayed ACLR Cohorts From Four Included Studies eFigure 6. Forest Plot of the Results of Re-Tear of Early ACLR Versus Elective Delayed ACLR eFigure 7. Forest Plot of the Results Infection of Early ACLR Versus Elective Delayed ACLR eFigure 8. Forest Plots Depicting the Extension Deficit and Flexion Deficit of Early ACLR Versus Elective Delayed ACLR After Redefinition eFigure 9. Forest Plot Depicting the Knee Laxity of Early ACLR Versus Elective Delayed ACLR After Redefinition eFigure 10. Forest Plot Depicting the Lysholm Score of Early ACLR Versus Elective Delayed ACL After Redefinition eFigure 11. Forest Plot Depicting the Tegner Score of Early ACLR Versus Elective Delayed ACLR After Redefinition eFigure 12. Forest Plots Depicting the IKDC Score and IKDC Rating Scale of Early ACLR Versus Elective Delayed ACLR After Redefinition eFigure 13. Forest Plot of the Results of Re-Tear of Early ACLR Versus Elective Delayed ACLR After Redefinition eFigure 14. Forest Plot of the Results Infection of Early ACLR Versus Elective Delayed ACLR After Redefinition [file jamanetwopen-e2242742-s001.pdf]

Supplemental Online Content

Shen X, Liu T, Xu S, et al. Optimal timing of anterior cruciate ligament reconstruction in patients with anterior cruciate ligament tear: a systematic review and meta-analysis. *JAMA Netw Open*. 2022;5(11):e2242742. doi:10.1001/jamanetworkopen.2022.42742

eMethods

eTable 1. Study Inclusion and Exclusion criteria

eTable 2. Risk of Bias of Assessment for the Included RCTs Using Cochrane Collaboration's Tool

eTable 3. Patient and Treatment Characteristics of Included Trials

eTable 4. Summary of Adverse Events After Early and Delayed ACLR in Included RCTs

eFigure 1. Forest Plot Depicting the Operative Time of Early ACLR Versus Elective Delayed ACLR

eFigure 2. Forest Plots Depicting the Extension Deficit and Flexion Deficit of Early ACLR Versus Elective Delayed ACLR

eFigure 3. Forest Plot Depicting the Tegner Score of Early ACLR Versus Elective Delayed ACLR

eFigure 4. Forest Plots Depicting the IKDC Score and IDKC Rating Scale of Early ACLR Versus Elective Delayed ACLR

eFigure 5. KOOS Subscales for Early ACLR and Elective Delayed ACLR Cohorts From Four Included Studies

eFigure 6. Forest Plot of the Results of Re-Tear of Early ACLR Versus Elective Delayed ACLR

eFigure 7. Forest Plot of the Results Infection of Early ACLR Versus Elective Delayed ACLR

eFigure 8. Forest Plots Depicting the Extension Deficit and Flexion Deficit of Early ACLR Versus Elective Delayed ACLR After Redefinition

eFigure 9. Forest Plot Depicting the Knee Laxity of Early ACLR Versus Elective Delayed ACLR After Redefinition

eFigure 10. Forest Plot Depicting the Lysholm Score of Early ACLR Versus Elective Delayed ACL After Redefinition

eFigure 11. Forest Plot Depicting the Tegner Score of Early ACLR Versus Elective Delayed ACLR After Redefinition

© 2022 Shen X et al. *JAMA Network Open.*

eFigure 12. Forest Plots Depicting the IKDC Score and IKDC Rating Scale of Early ACLR Versus Elective Delayed ACLR After Redefinition

eFigure 13. Forest Plot of the Results of Re-Tear of Early ACLR Versus Elective Delayed ACLR After Redefinition

eFigure 14. Forest Plot of the Results Infection of Early ACLR Versus Elective Delayed ACLR After Redefinition

This supplemental material has been provided by the authors to give readers additional information about their work.

© 2022 Shen X et al. *JAMA Network Open.*

eMethods

Search strategy and trial selection

The protocol for the systematic review is registered on PROSPERO (CRD42018089972). The PubMed, Cochrane Library, and Web of Science databases were systematically searched in September 2022. Using a search strategy for combined terms (((anterior cruciate ligament OR ACL) AND (reconstruction OR surgery OR repair)) AND (early OR acute)) AND (delayed OR chronic). The references in the included articles were further reviewed to identify additional studies.

The inclusion and exclusion criteria were reported in eTable 1 in Supplement. To ensure that the selected articles met the specified inclusion criteria, the titles and abstracts of the studies were independently reviewed by two authors (XYS and BC) in a blinded manner. Any disagreements on trial inclusion and data were resolved through discussion and consensus with the participation of a senior reviewer (JLX).

Data extraction

The extracted data included the following: study design, randomization, definition of surgery timing, inclusion/exclusion criteria, operative technique, rehabilitation protocols, and follow-up duration. The following participant and surgical characteristics were also collected: participants' sample size, age, sex, graft type, associated lesions, injury mechanisms, injury-to-surgery time, and rehabilitation principle. The selected clinical outcomes took into account the most commonly used outcome measures in recent publications.

Statistical analysis

To evaluate the outcomes of early *vs.* elective delayed ACLR after different follow-up durations, we recorded the data given for all follow-up time points. The included trials were grouped according to their follow-up durations as follows: 6 months, 1 year, 2 years, and 5 years. If the relevant outcomes were reported at multiple follow-up time points, the data were analyzed separately for each time point. When same RCTs were included in subgroup analyses of different follow-up duration, only subtotals were calculated. All eligible studies were included in the meta-analyses and subgroup analyses, as applicable.

To estimate the standardized mean difference (sMD), we calculated the mean and standard deviation (SD) values. If the mean and SD data were not provided in the included studies, the sMD was calculated using the P value and sample size. The I^2 statistic was considered to evaluate the data

© 2022 Shen X et al. *JAMA Network Open.*

for heterogeneity among studies and confirm the appropriateness of pooling among groups. Clinical heterogeneity was assumed present, a random-effects model was preferred.

© 2022 Shen X et al. *JAMA Network Open.*

eTable 1. Study Inclusion and Exclusion Criteria

**Inclusion Criteria**	**Exclusion Criteria**
Randomized clinical Trial	Non-English articles
Clinical or functional outcomes and adverse complications associated with early versus elective delayed ACLR	Case series and reviews
	Not compare clinical outcomes between early and delayed ACLR
	Not clearly define specific cutoff points for early and elective delayed surgery

Note: ACLR, anterior cruciate ligament reconstruction.

© 2022 Shen X et al. *JAMA Network Open.*

eTable 2. Risk of Bias of Assessment for the Included RCTs Using Cochrane Collaboration's Tool

Author (Year)	Sequence generation	Allocation concealment	Blinding	Incomplete outcome data	Selective outcome report	Free of other bias
**Meighan et al.[19] (2003)**	✓	✓	✓	?	✓	✓
**Bottoni et al.[20] (2008)**	✓	✓	✓	✓	✓	✓
**Raviraj et al.[21] (2010)**	✓	✓	✓	✗	?	?
**Frobell et al.[22] (2010)**	✓	✓	✓	✓	✓	✓
**Frobell et al.[23] (2013)**	✓	✓	✓	✓	✓	✓
**Chen et al.[24] (2015)**	✓	✓	✓	✓	?	?
**Manandhar et al.[25] (2018)**	✗	✗	✓	✓	✓	?
**Eriksson et al.[26] (2018)**	✓	✓	✓	✓	✓	?
**von Essen et al.[16] (2020)**	✓	✓	✓	✓	✓	?
**von Essen et al.[27] (2020)**	✓	✓	✓	✓	✓	?
**Reijman et al.[17] (2021)**	✓	✓	✓	✓	✓	✓

Note: ✓= Low risk of bias, ? = Unclear risk of bias, ✗= High risk of bias.

© 2022 Shen X et al. *JAMA Network Open.*

eTable 3. Patient and Treatment Characteristics of Included Trials

Author (Year)	Timing of ACLR	No. of Patients	Age	M/F	Graft type	Associated lesions		Mechanisms of injury	Injury to surgery	Operation time (min)
						Meniscal injury	Chondral injuries			
**Meighan et al.[19] (2003)**	Early	13	21 (15-35)	28/3	hamstring	3	NA	18 football, 6 rugby, 4 basketball	NA	67
	Delayed	18			hamstring	4	NA		NA	74
**Bottoni et al.20 (2008)**	Early	35	26.4 (18-40)	29/6	hamstring	32	9	49 sports, 13 falls, 4 training accidents, 4 vehicle accidents	9.0 ± 4.4	64.0 ± 25.5
	Delayed	35	27.5(19-43)	29/6	hamstring	24	5		84.8 ± 38.2	61.5 ± 23.9
**Raviraj et al.[21] (2010)**	Early	51	31.6 ± 5.3	25/26	hamstring	38	29	23 fall, 21 sports injury, 7 traffic accidents	7 (2-14)	64.9 ± 7.8
	Delayed	48	31.2 ± 5.3	26/22	hamstring	35	31	15 fall, 24 sports injury, 9 traffic accidents	32 (29-42)	64.2 ± 7.8
**Frobell et al.[22] (2010)**	Early	62	26.3 ± 5.1	48/12	36 hamstring, 25 BPTB	39	NA	35 soccer, 9 Alphine skiing, 7 floor hockey, 11 others	23.4 ± 9.5	NA
	Delayed	23	25.8 ± 4.7	16/7	10 hamstring, 13 BPTB	30	NA	42 soccer, 7 Alphine skiing, 2 floor hockey, 8 others	347 ± 124	NA
	Rehabilitation	36		23/13	NA				NA	
**Frobell et al.[23] (2013)**	Early	62	26.6 ± 5.1	47/12	36 hamstring, 25 BPTB	NA	NA	35 soccer, 9 Alphine skiing, 7 floor hockey, 11 others	23.4 ± 9.5	NA
	Delayed	30	25.2 ± 4.5	19/11	15 hamstring, 15 BPTB	NA	NA	42 soccer, 7 Alphine skiing, 2 floor hockey, 8 others	867 (743-1695)	NA
	Rehabilitation	29	26.4 ± 4.9	20/9	NA	NA	NA		NA	NA
**Chen et al.24 (2015)**	Acute	27	29.4 ± 5.8	15/12	LARS graft	NA	NA	NA	5.4 w (3–7)	NA
	Chronic	28	31.9 ± 7.0	11/17	LARS graft	NA	NA	NA	7.2 m (6–11)	NA
**Manandhara**	Early	53	30 (18-55)	83/21	hamstring	22	10	73 sports injury, 26 road traffic	11.20 (4-21)	NA

© 2022 Shen X et al. *JAMA Network Open.*

**et al.[25] (2018)**	Delayed	51			hamstring	34	28	accidents, 5 others	48 (42-60)	NA
**Eriksson et al.[26] (2018)**	Early	33	27.7±6.5	23/10	hamstring	20	10	13 soccer, 6 indoor floorball 7 Alphine skiing, 1 handball, 3 wrestling, 2 gymnastics, 1 Dance	5 ± 2	93 ± 20
	Delayed	35	26.1±5.7	24/11	hamstring	12	4	13 soccer, 10 indoor floorball, 3 Alphine skiing, 4 handball, 1 Ice hockey, 1 football, 1badminton, 1basketball, 1 tennis	55 ± 8	83 ± 18
**von Essen et al.[16] (2020)**	Early	33	27.7±6.5	23/10	hamstring	20	10	13 soccer, 6 indoor floorball, 7 Alphine skiing, 7 other sports activity	5 ± 2	93 ± 20
	Delayed	35	26.1±5.7	24/11	hamstring	12	4	13 soccer, 10 indoor floorball, 3 Alphine skiing, 9 other sports activity	55 ± 8	83 ± 18
**von Essen et al.[27] (2020)**	Early	34	27.7 ± 6.5	24/10	hamstring	20	10	14 soccer, 6 indoor floorball, 7 Alphine skiing, 7 other sports activity	5 ± 2	93 ± 20
	Delayed	35	26.1±5.7	24/11	hamstring	12	4	13 soccer, 10 indoor floorball, 3 Alphine skiing, 9 other sports activity	55 ± 8	83 ± 18
**Reijman et al.[17] (2021)**	Early	85	31.2±10.3	36/49	78hamstring, 4BPTB	38	23	NA	39.0 (25.5-53.0)	NA
	Rehabilitation with optional delayed	82	31.4±10.7	31/51	41ACLR, (38hamstring, 3BPTB)	37	16	NA	40.5 (29.8-52.5)	NA

Note: ACLR, anterior cruciate ligament reconstruction; BPTB, bone-patellar tendon-bone; NA, not available; LARS, ligament advanced reinforcement system.

© 2022 Shen X et al. *JAMA Network Open.*

eTable 4. Summary of Adverse Events After Early and Delayed ACLR in Included RCTs

**Author (Year)**	Adverse Events	
	**Early ACLR**	**Elective delayed ACLR**
**Meighan et al.[19] (2003)**	2 deep vein thrombosis; 1 wound infection; 1 extension deficit; 1 painful tibial fixation screw; 1 knee stiffness	1 knee stiffness; 1 subjective instability; 1 retear
**Bottoni et al.[20] (2008)**	1 intra-articular infection; 1 retear; one 5°-10° loss of extension; one > 10°loss of flexion; five 5°-10° loss of flexion	1 retear; One >10° loss of extension; two 5°-10° loss of flexion; five 5°-10 loss of flexion
**Raviraj et al.[21] (2010)**	2 superficial wound infection	1 pain
**Frobell et al.[22] (2010)**	2 subjective or clinical instability; 1 meniscal signs and symptoms; 6 pain, swelling, or both; 4 decreased ROM;1 extension deficit; 1 arthrofibrosis; 3 retear	19 subjective or clinical instability; 13 meniscal signs and symptoms; 3 pain, swelling, or both; 1 decreased ROM; 1 retear
**Frobell et al.[23] (2013)**	19 radiographic osteoarthritis; 3 retear	10 radiographic osteoarthritis; 1 retear
**Chen et al.[24] (2015)**	1 mild arthrofibrosis; 1 arthralgia due to loosen screw	1 mild arthrofibrosis
**Manandhara et al.[25] (2018)**	1 infection	None
**Eriksson et al.[26] (2018)**	Seven > 5° extension defects	Thirteen > 5° extension defects
**von Essen et al.[16] (2020)**	1 retear; four > 5° extension defects	1 retear; five > 5° extension defects
**von Essen et al.[27] (2020)**	NA	NA
**MaxReij et al.[17] (2021)**	4 retear; 3 ruptures of contralateral ACL; 1 tibial screw events; 4 meniscal tear; 2 extension deficit	2 retear; 1 rupture of contralateral ACL; 2 tibial screw events; 3 meniscal tear; 4 extension deficit

Note: ACLR, anterior cruciate ligament reconstruction; ROM, range of motion; NA, not available.

© 2022 Shen X et al. *JAMA Network Open.*

eFigure 1. Forest Plot Depicting the Operative Time of Early ACLR Versus Elective Delayed ACLR

	Early ACLR			Elective delayed ACLR				Mean Difference
Study or Subgroup	Mean	SD	Total	Mean	SD	Total	Weight	IV, Random, 95% CI
Bottoni 2008	64	25.5	34	61.5	23.9	35	15.7%	2.50 [-9.17, 14.17]
Eriksson 2018	93	20	33	83	18	35	21.3%	10.00 [0.94, 19.06]
Raviraj 2010	64.9	7.8	51	64.2	7.8	48	41.5%	0.70 [-2.37, 3.77]
von Essen (12m) 2020	93	20	34	83	18	35	21.5%	10.00 [1.01, 18.99]
**Total (95% CI)**			**152**			**153**	**100.0%**	**4.97 [-0.68, 10.61]**

Heterogeneity: $Tau^2 = 17.52$; $Chi^2 = 6.62$, df = 3 (P = 0.08); $I^2 = 55\%$
Test for overall effect: Z = 1.72 (P = 0.08)

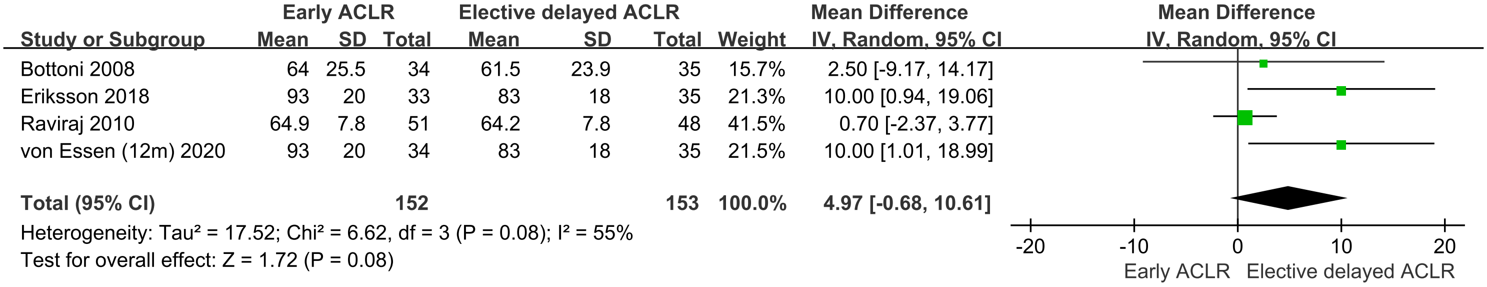

© 2022 Shen X et al. *JAMA Network Open.*

eFigure 2. Forest Plots Depicting the Extension Deficit and Flexion Deficit of Early ACLR Versus Elective Delayed ACLR

	Early ACLR			Elective delayed ACLR				Mean Difference
Study or Subgroup	Mean	SD	Total	Mean	SD	Total	Weight	IV, Random, 95% CI
**2.1.1 Follow-up 6 months**								
Eriksson 2018	3	3	33	4	3.5	35	100.0%	-1.00 [-2.55, 0.55]
**Subtotal (95% CI)**			**33**			**35**	**100.0%**	**-1.00 [-2.55, 0.55]**
Heterogeneity: Not applicable								
Test for overall effect: Z = 1.27 (P = 0.21)								
**2.1.2 Follow-up 1 year**								
Bottoni 2008	0.6	1.6	34	1.5	4.2	35	43.9%	-0.90 [-2.39, 0.59]
von Essen (12m) 2020	2	2.1	31	3	3.3	35	56.1%	-1.00 [-2.32, 0.32]
**Subtotal (95% CI)**			**65**			**70**	**100.0%**	**-0.96 [-1.94, 0.03]**
Heterogeneity: Tau² = 0.00; Chi² = 0.01, df = 1 (P = 0.92); I² = 0%								
Test for overall effect: Z = 1.90 (P = 0.06)								
**2.1.3 Follow-up 2 years**								
von Essen (24m) 2020	1.6	3	28	1.3	2.5	29	100.0%	0.30 [-1.14, 1.74]
**Subtotal (95% CI)**			**28**			**29**	**100.0%**	**0.30 [-1.14, 1.74]**
Heterogeneity: Not applicable								
Test for overall effect: Z = 0.41 (P = 0.68)								

Mean Difference
IV, Random, 95% CI

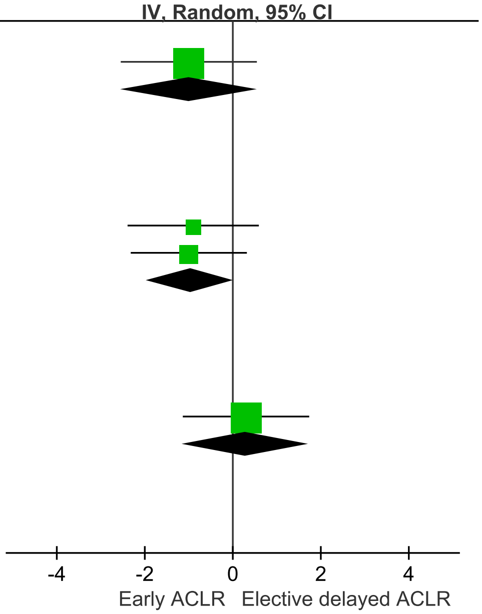

© 2022 Shen X et al. *JAMA Network Open.*

Study or Subgroup	Early ACLR Mean	Early ACLR SD	Early ACLR Total	Elective delayed ACLR Mean	Elective delayed ACLR SD	Elective delayed ACLR Total	Weight	Mean Difference IV, Random, 95% CI
**2.2.1 Follow-up 6 months**								
Eriksson 2018	4	5.4	33	5	5.4	35	100.0%	-1.00 [-3.57, 1.57]
**Subtotal (95% CI)**			**33**			**35**	**100.0%**	**-1.00 [-3.57, 1.57]**
Heterogeneity: Not applicable								
Test for overall effect: Z = 0.76 (P = 0.45)								
**2.2.2 Follow-up 1 year**								
Bottoni 2008	2.1	3.9	34	2.6	4.8	35	30.6%	-0.50 [-2.56, 1.56]
von Essen (12m) 2020	1.8	2.2	31	3.2	3.4	35	69.4%	-1.40 [-2.77, -0.03]
**Subtotal (95% CI)**			**65**			**70**	**100.0%**	**-1.13 [-2.26, 0.01]**
Heterogeneity: Tau² = 0.00; Chi² = 0.51, df = 1 (P = 0.48); I² = 0%								
Test for overall effect: Z = 1.94 (P = 0.05)								
**2.2.3 Follow-up 2 years**								
von Essen (24m) 2020	1.75	2.8	28	2.8	4.1	29	100.0%	-1.05 [-2.87, 0.77]
**Subtotal (95% CI)**			**28**			**29**	**100.0%**	**-1.05 [-2.87, 0.77]**
Heterogeneity: Not applicable								
Test for overall effect: Z = 1.13 (P = 0.26)								

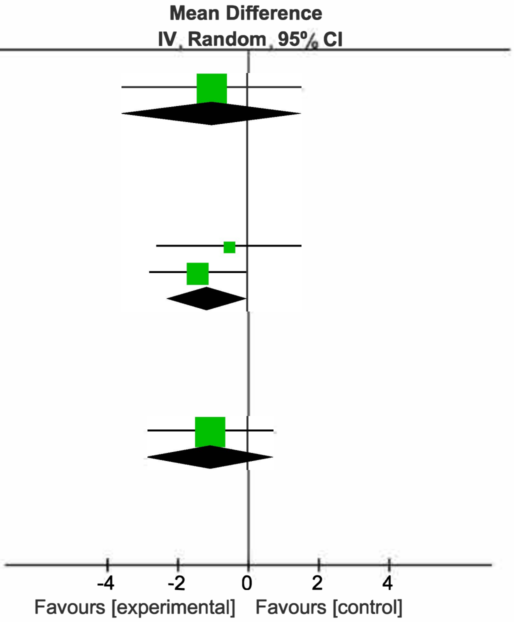

© 2022 Shen X et al. *JAMA Network Open*.

eFigure 3. Forest Plot Depicting the Tegner Score of Early ACLR Versus Elective Delayed ACLR

	Elective delayed ACLR			Early ACLR				Mean Difference
Study or Subgroup	Mean	SD	Total	Mean	SD	Total	Weight	IV, Random, 95% CI
**3.1.1 Follow-up 6 months**								
Manandhar 2018	4.15	1.45	53	3.72	1.34	54	100.0%	0.43 [-0.10, 0.96]
**Subtotal (95% CI)**			**53**			**54**	**100.0%**	**0.43 [-0.10, 0.96]**
Heterogeneity: Not applicable								
Test for overall effect: Z = 1.59 (P = 0.11)								
**3.1.2 Follow-up 1 year**								
Chen 2015	6.3	1.1	27	6.1	0.9	28	100.0%	0.20 [-0.33, 0.73]
**Subtotal (95% CI)**			**27**			**28**	**100.0%**	**0.20 [-0.33, 0.73]**
Heterogeneity: Not applicable								
Test for overall effect: Z = 0.74 (P = 0.46)								
**3.1.3 Follow-up 5 years**								
Chen 2015	6.3	1.3	27	6.3	1.2	28	100.0%	0.00 [-0.66, 0.66]
**Subtotal (95% CI)**			**27**			**28**	**100.0%**	**0.00 [-0.66, 0.66]**
Heterogeneity: Not applicable								
Test for overall effect: Z = 0.00 (P = 1.00)								

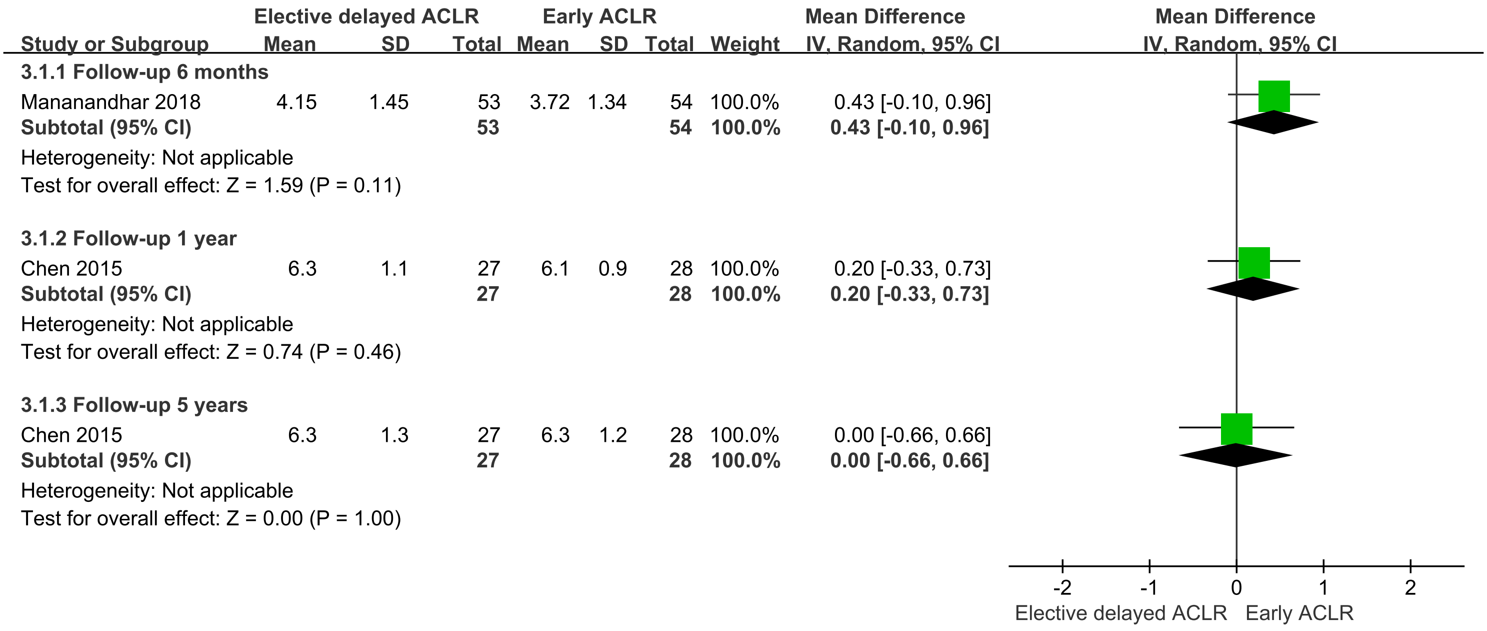

© 2022 Shen X et al. *JAMA Network Open.*

eFigure 4. Forest Plots Depicting the IKDC Score and IDKC Rating Scale of Early ACLR Versus Elective Delayed ACLR

Study or Subgroup	Early ACLR Mean	Early ACLR SD	Early ACLR Total	Elective delayed ACLR Mean	Elective delayed ACLR SD	Elective delayed ACLR Total	Weight	Mean Difference IV, Random, 95% CI
**4.1.1 Follow-up 6 months**								
Mananandhar 2018	69.68	8.14	53	67.14	6.08	51	10.4%	2.54 [-0.21, 5.29]
Reijman 2021	69.6	3.1	83	66.8	3	80	89.6%	2.80 [1.86, 3.74]
**Subtotal (95% CI)**			**136**			**131**	**100.0%**	**2.77 [1.89, 3.66]**
Heterogeneity: Tau² = 0.00; Chi² = 0.03, df = 1 (P = 0.86); I² = 0%								
Test for overall effect: Z = 6.13 (P < 0.00001)								
**Total (95% CI)**			**136**			**131**	**100.0%**	**2.77 [1.89, 3.66]**
Heterogeneity: Tau² = 0.00; Chi² = 0.03, df = 1 (P = 0.86); I² = 0%								
Test for overall effect: Z = 6.13 (P < 0.00001)								
Test for subgroup differences: Not applicable								

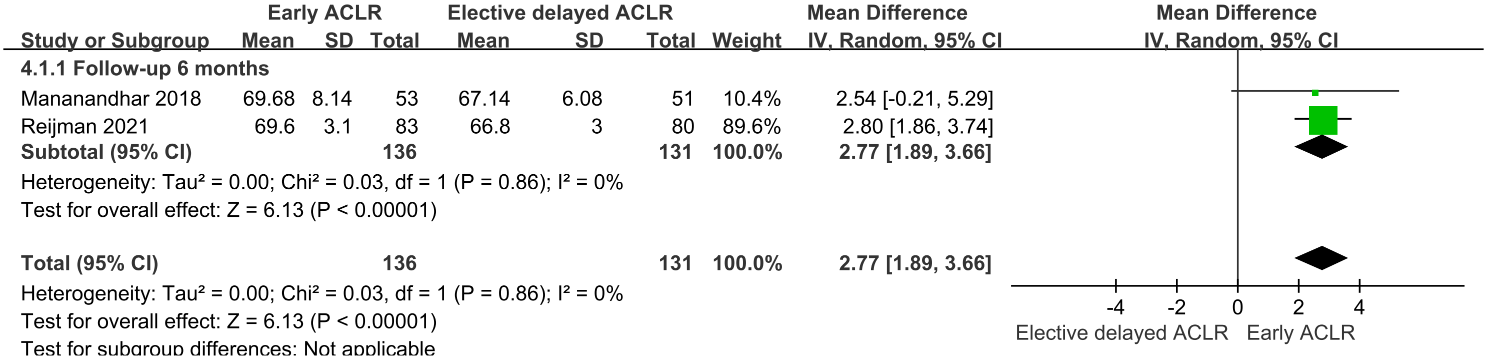

© 2022 Shen X et al. *JAMA Network Open.*

Study or Subgroup	Early ACLR Events	Early ACLR Total	Elective delayed ACLR Events	Elective delayed ACLR Total	Weight	Odds Ratio M-H, Random, 95% CI
**4.2.1 Follow-up 6 months**						
Eriksson 2018	27	33	24	34	100.0%	1.88 [0.59, 5.93]
**Subtotal (95% CI)**		**33**		**34**	**100.0%**	**1.88 [0.59, 5.93]**
Total events	27		24			
Heterogeneity: Not applicable						
Test for overall effect: Z = 1.07 (P = 0.28)						
**4.2.2 Follow-up 1 year**						
Meighan 2003	11	13	15	18	29.7%	1.10 [0.16, 7.74]
von Essen (12m) 2020	26	31	27	34	70.3%	1.35 [0.38, 4.79]
**Subtotal (95% CI)**		**44**		**52**	**100.0%**	**1.27 [0.44, 3.67]**
Total events	37		42			
Heterogeneity: Tau² = 0.00; Chi² = 0.03, df = 1 (P = 0.86); I² = 0%						
Test for overall effect: Z = 0.44 (P = 0.66)						
**4.2.3 Follow-up 2 year**						
von Essen (24m) 2020	24	27	28	28	100.0%	0.12 [0.01, 2.50]
**Subtotal (95% CI)**		**27**		**28**	**100.0%**	**0.12 [0.01, 2.50]**
Total events	24		28			
Heterogeneity: Not applicable						
Test for overall effect: Z = 1.36 (P = 0.17)						
**4.2.4 Follow-up 5 year**						
Chen 2015	26	27	26	28	100.0%	2.00 [0.17, 23.44]
**Subtotal (95% CI)**		**27**		**28**	**100.0%**	**2.00 [0.17, 23.44]**
Total events	26		26			
Heterogeneity: Not applicable						
Test for overall effect: Z = 0.55 (P = 0.58)						

Odds Ratio M-H, Random, 95% CI

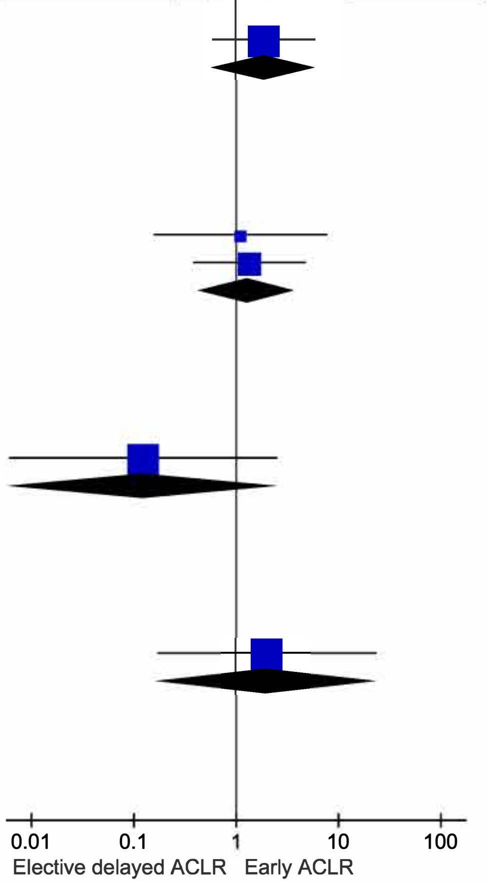

© 2022 Shen X et al. *JAMA Network Open.*

eFigure 5. KOOS Subscales for Early ACLR and Elective Delayed ACLR Cohorts From Four Included Studies

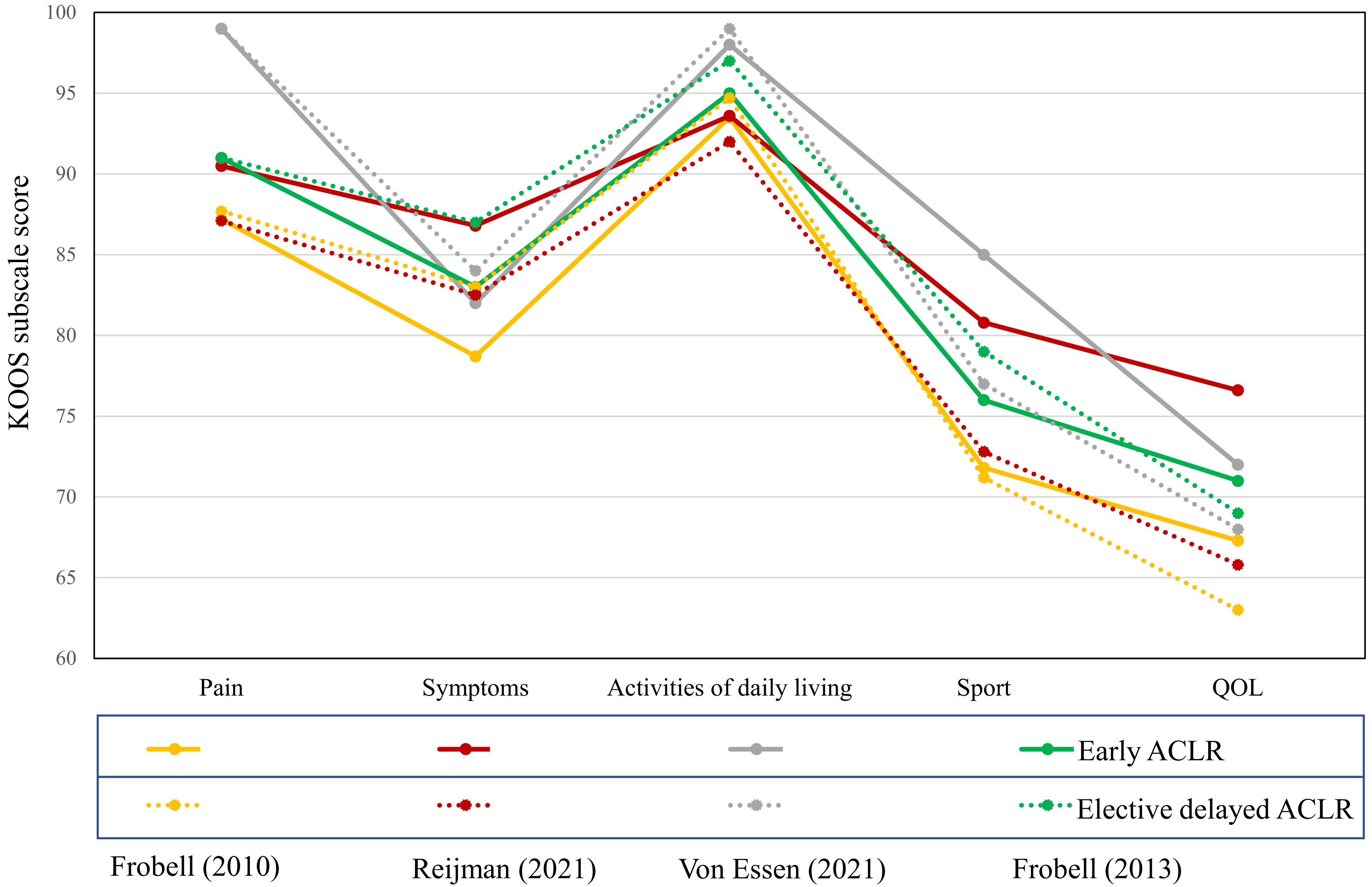

© 2022 Shen X et al. *JAMA Network Open.*

eFigure 6. Forest Plot of the Results of Re-Tear of Early ACLR Versus Elective Delayed ACLR

	Early ACLR		Elective delayed ACLR			Odds Ratio	
Study or Subgroup	Events	Total	Events	Total	Weight	M-H, Random, 95% CI	Year
Meighan 2003	0	13	1	18	10.7%	0.43 [0.02, 11.47]	2003
Bottoni 2008	1	34	1	35	14.5%	1.03 [0.06, 17.16]	2008
Frobell 2010	3	62	1	59	21.8%	2.95 [0.30, 29.18]	2010
Chen 2015	0	27	0	28		Not estimable	2015
von Essen (12m) 2020	1	34	1	35	14.5%	1.03 [0.06, 17.16]	2020
Reijman 2021	4	85	2	82	38.5%	1.98 [0.35, 11.09]	2021
**Total (95% CI)**		**255**		**257**	**100.0%**	**1.52 [0.52, 4.43]**	
Total events	9		6				

Heterogeneity: Tau² = 0.00; Chi² = 1.12, df = 4 (P = 0.89); I² = 0%

Test for overall effect: Z = 0.76 (P = 0.44)

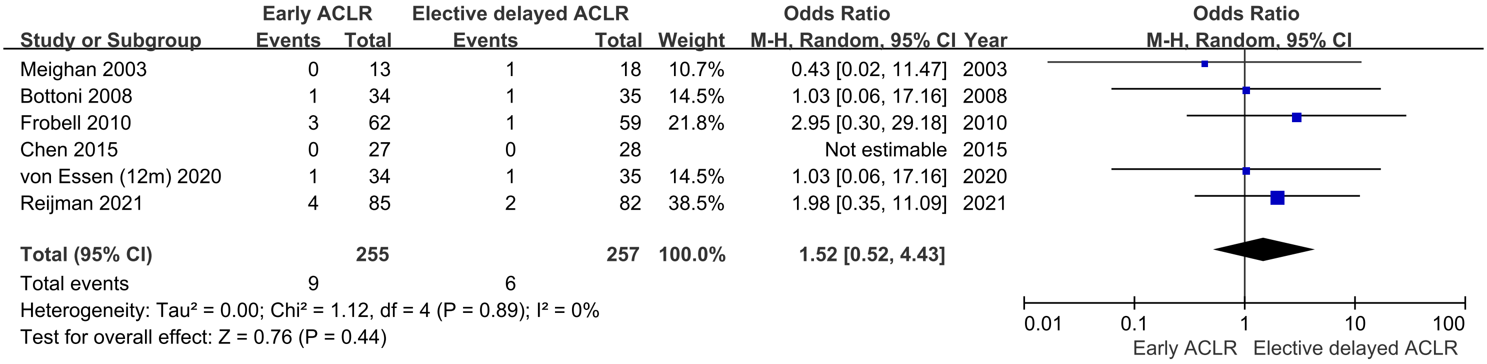

© 2022 Shen X et al. *JAMA Network Open.*

eFigure 7. Forest Plot of the Results Infection of Early ACLR Versus Elective Delayed ACLR

	Early ACLR		Elective Delayed ACLR			Odds Ratio	
**Study or Subgroup**	**Events**	**Total**	**Events**	**Total**	**Weight**	**M-H, Random, 95% CI**	**Year**
Meighan 2003	1	13	0	18	23.7%	4.44 [0.17, 118.00]	2003
Bottoni 2008	1	34	0	35	24.4%	3.18 [0.13, 80.79]	2008
Raviraj 2010	2	51	0	48	27.3%	4.90 [0.23, 104.70]	2010
Chen 2015	0	27	0	28		Not estimable	2015
Mananandhar 2018	1	53	0	51	24.6%	2.94 [0.12, 73.92]	2018
**Total (95% CI)**		**178**		**180**	**100.0%**	**3.80 [0.77, 18.79]**	
Total events	5		0				

Heterogeneity: Tau² = 0.00; Chi² = 0.07, df = 3 (P = 1.00); I² = 0%
Test for overall effect: Z = 1.64 (P = 0.10)

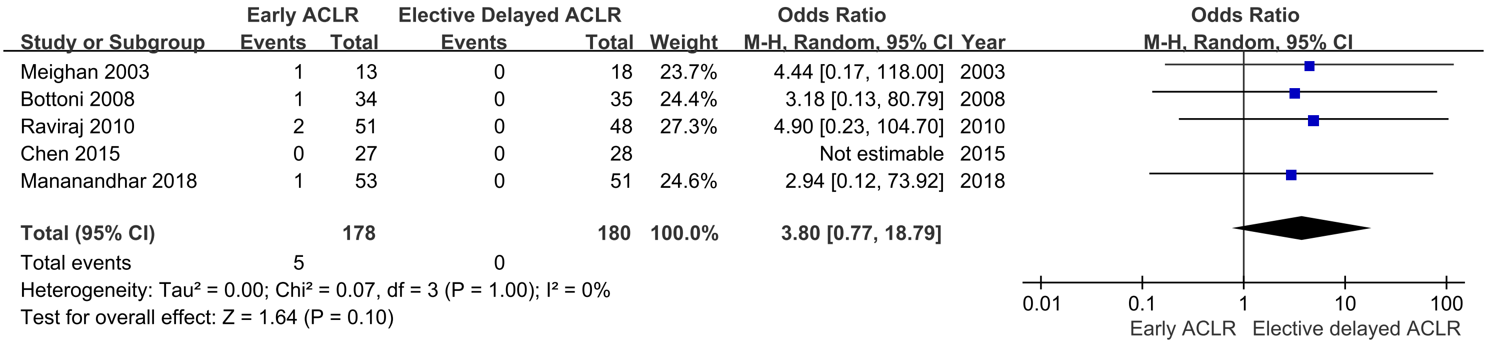

© 2022 Shen X et al. *JAMA Network Open.*

eFigure 8. Forest Plots Depicting the Extension Deficit and Flexion Deficit of Early ACLR Versus Elective Delayed ACLR After Redefinition

Study or Subgroup	Early ACLR Mean	Early ACLR SD	Early ACLR Total	Delayed ACLR Mean	Delayed ACLR SD	Delayed ACLR Total	Weight	Mean Difference IV, Random, 95% CI
**8.1.1 Follow-up 6 months**								
Eriksson 2018	3	3	33	4	3.5	35	100.0%	-1.00 [-2.55, 0.55]
**Subtotal (95% CI)**			**33**			**35**	**100.0%**	**-1.00 [-2.55, 0.55]**
Heterogeneity: Not applicable								
Test for overall effect: Z = 1.27 (P = 0.21)								
**8.1.2 Follow-up 1 year**								
Bottoni 2008	0.6	1.6	34	1.5	4.2	35	43.9%	-0.90 [-2.39, 0.59]
von Essen (12m) 2020	2	2.1	31	3	3.3	35	56.1%	-1.00 [-2.32, 0.32]
**Subtotal (95% CI)**			**65**			**70**	**100.0%**	**-0.96 [-1.94, 0.03]**
Heterogeneity: Tau² = 0.00; Chi² = 0.01, df = 1 (P = 0.92); I² = 0%								
Test for overall effect: Z = 1.90 (P = 0.06)								
**8.1.3 Follow-up 2 years**								
von Essen (24m) 2020	1.6	3	28	1.3	2.5	29	100.0%	0.30 [-1.14, 1.74]
**Subtotal (95% CI)**			**28**			**29**	**100.0%**	**0.30 [-1.14, 1.74]**
Heterogeneity: Not applicable								
Test for overall effect: Z = 0.41 (P = 0.68)								

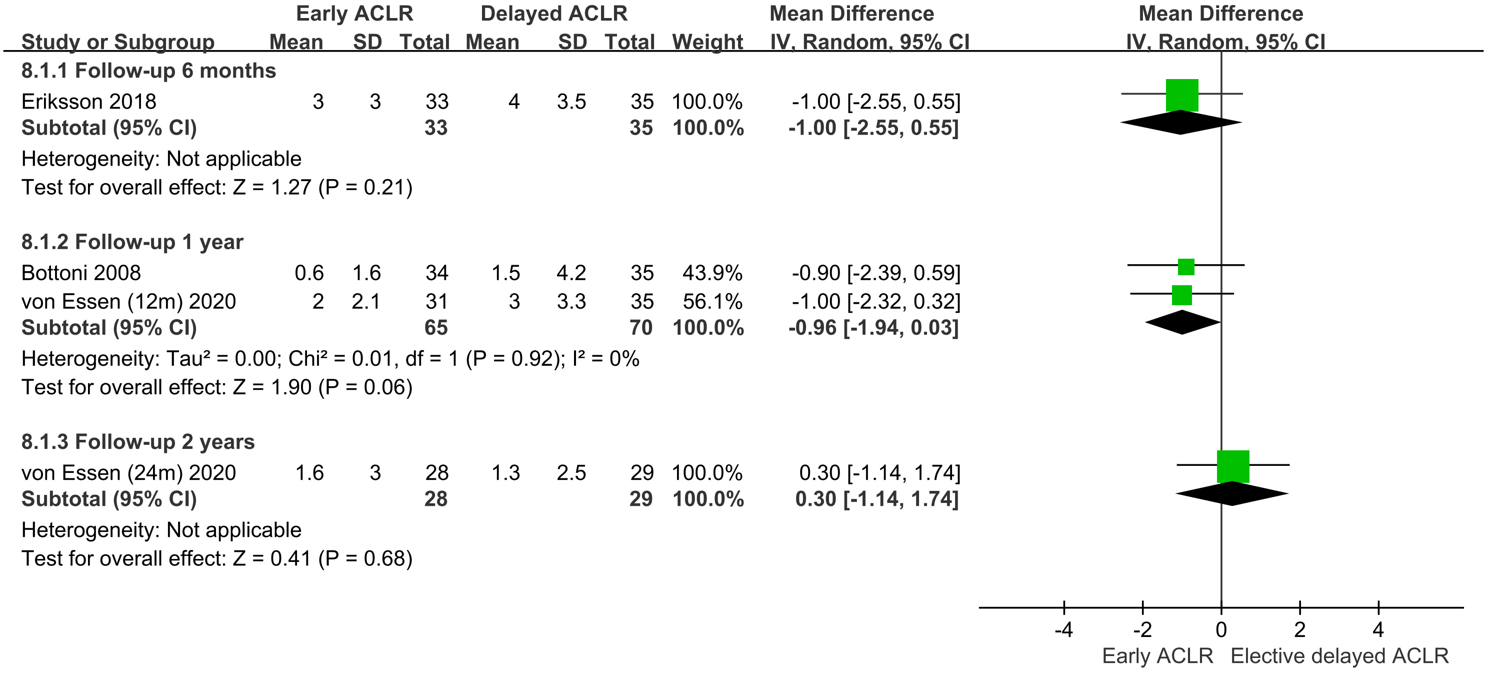

© 2022 Shen X et al. *JAMA Network Open.*

Study or Subgroup	Early ACLR Mean	Early ACLR SD	Early ACLR Total	Delayed ACLR Mean	Delayed ACLR SD	Delayed ACLR Total	Weight	Mean Difference IV, Random, 95% CI
**8.2.1 Follow-up 6 months**								
Eriksson 2018	4	5.4	33	5	5.4	35	100.0%	-1.00 [-3.57, 1.57]
**Subtotal (95% CI)**			**33**			**35**	**100.0%**	**-1.00 [-3.57, 1.57]**
Heterogeneity: Not applicable								
Test for overall effect: Z = 0.76 (P = 0.45)								
**8.2.2 Follow-up 1 year**								
Bottoni 2008	2.1	3.9	34	2.6	4.8	35	30.6%	-0.50 [-2.56, 1.56]
von Essen (12m) 2020	1.8	2.2	31	3.2	3.4	35	69.4%	-1.40 [-2.77, -0.03]
**Subtotal (95% CI)**			**65**			**70**	**100.0%**	**-1.13 [-2.26, 0.01]**
Heterogeneity: Tau² = 0.00; Chi² = 0.51, df = 1 (P = 0.48); I² = 0%								
Test for overall effect: Z = 1.94 (P = 0.05)								
**8.2.3 Folluw-up 2 years**								
von Essen (24m) 2020	1.75	2.8	28	2.8	4.1	29	100.0%	-1.05 [-2.87, 0.77]
**Subtotal (95% CI)**			**28**			**29**	**100.0%**	**-1.05 [-2.87, 0.77]**
Heterogeneity: Not applicable								
Test for overall effect: Z = 1.13 (P = 0.26)								

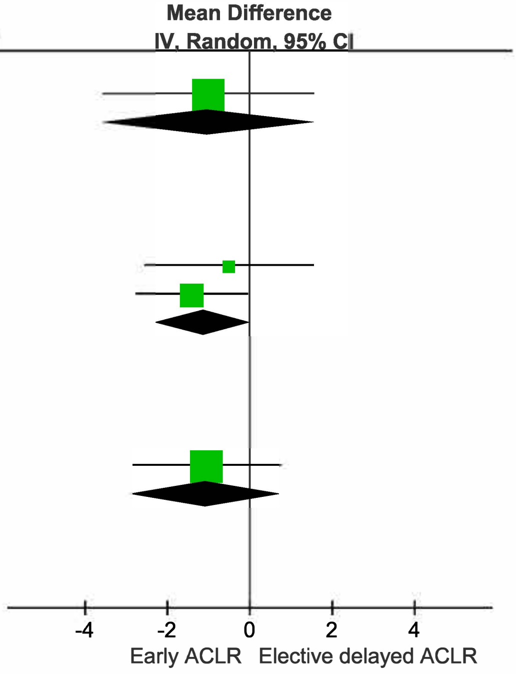

© 2022 Shen X et al. *JAMA Network Open.*

eFigure 9. Forest Plot Depicting the Knee Laxity of Early ACLR Versus Elective Delayed ACLR After Redefinition

Study or Subgroup	Early ACLR Mean	SD	Total	Delayed ACLR Mean	SD	Total	Weight	Std. Mean Difference IV, Random, 95% CI
**9.1.1 Follow-up 6 months**								
Eriksson 2018	2.3	1.4	32	1.8	1.2	33	100.0%	0.38 [-0.11, 0.87]
**Subtotal (95% CI)**			**32**			**33**	**100.0%**	**0.38 [-0.11, 0.87]**
Heterogeneity: Not applicable								
Test for overall effect: Z = 1.51 (P = 0.13)								
**9.1.2 Follow-up 1 year**								
Bottoni 2008	1.2	1.8	34	0.88	1.1	34	49.5%	0.21 [-0.26, 0.69]
von Essen (24m) 2020	2	1.4	34	1.9	1.2	35	50.5%	0.08 [-0.40, 0.55]
**Subtotal (95% CI)**			**68**			**69**	**100.0%**	**0.14 [-0.19, 0.48]**
Heterogeneity: Tau² = 0.00; Chi² = 0.16, df = 1 (P = 0.69); I² = 0%								
Test for overall effect: Z = 0.84 (P = 0.40)								
**9.1.3 Follow-up 2 year**								
von Essen (24m) 2020	1.8	1.5	28	2	1.5	29	100.0%	-0.13 [-0.65, 0.39]
**Subtotal (95% CI)**			**28**			**29**	**100.0%**	**-0.13 [-0.65, 0.39]**
Heterogeneity: Not applicable								
Test for overall effect: Z = 0.50 (P = 0.62)								

Std. Mean Difference
IV, Random, 95% CI

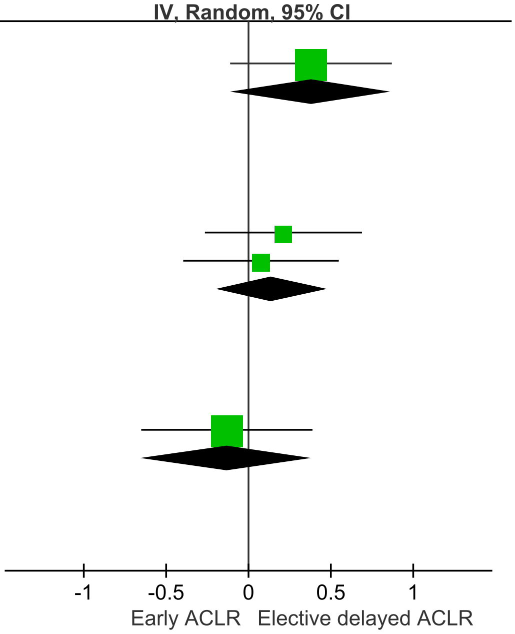

© 2022 Shen X et al. *JAMA Network Open.*

eFigure 10. Forest Plot Depicting the Lysholm Score of Early ACLR Versus Elective Delayed ACL After Redefinition

	Early ACLR			Delayed ACLR				Mean Difference
Study or Subgroup	Mean	SD	Total	Mean	SD	Total	Weight	IV, Random, 95% CI
**10.1.1 Follow-up 6 months**								
Eriksson 2018	76	16.2	33	79	15.2	35	39.3%	-3.00 [-10.48, 4.48]
Reijman 2021	86.6	3.5	83	81.4	1.3	80	60.7%	5.20 [4.39, 6.01]
**Subtotal (95% CI)**			**116**			**115**	**100.0%**	**1.98 [-5.87, 9.83]**
Heterogeneity: Tau² = 26.26; Chi² = 4.57, df = 1 (P = 0.03); I² = 78%								
Test for overall effect: Z = 0.49 (P = 0.62)								
**10.1.2 Follow-up 1 year**								
Reijman 2021	90.3	1.3	85	86.2	1.4	82	50.3%	4.10 [3.69, 4.51]
von Essen (24m) 2020	87	1.8	34	88	1.7	35	49.7%	-1.00 [-1.83, -0.17]
**Subtotal (95% CI)**			**119**			**117**	**100.0%**	**1.56 [-3.43, 6.56]**
Heterogeneity: Tau² = 12.89; Chi² = 117.35, df = 1 (P < 0.00001); I² = 99%								
Test for overall effect: Z = 0.61 (P = 0.54)								
**10.1.3 Follow-up 2 years**								
Reijman 2021	90.6	2.7	83	87.1	2.7	80	53.3%	3.50 [2.67, 4.33]
von Essen (24m) 2020	88.05	2.4	28	86.46	2.5	29	46.7%	1.59 [0.32, 2.86]
**Subtotal (95% CI)**			**111**			**109**	**100.0%**	**2.61 [0.74, 4.48]**
Heterogeneity: Tau² = 1.52; Chi² = 6.08, df = 1 (P = 0.01); I² = 84%								
Test for overall effect: Z = 2.74 (P = 0.006)								

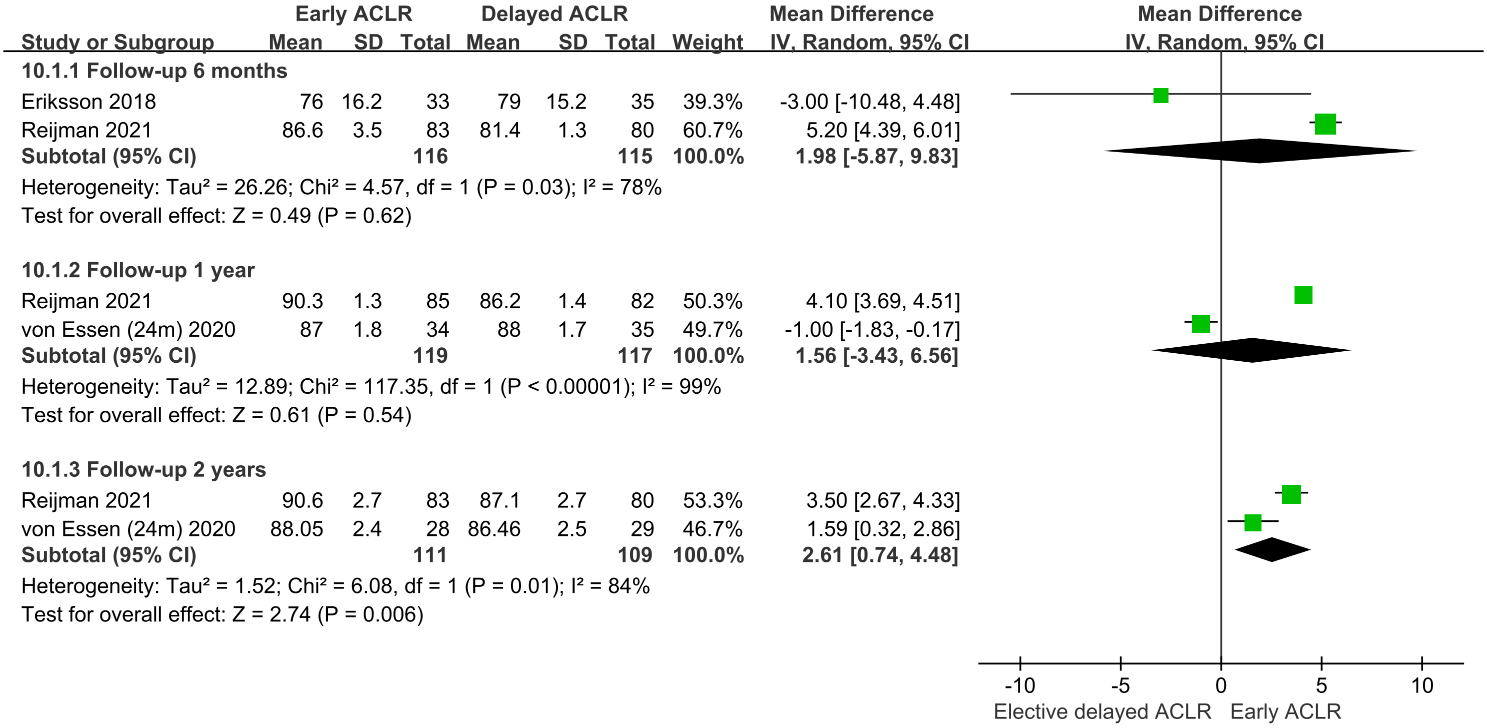

© 2022 Shen X et al. *JAMA Network Open*.

eFigure 11. Forest Plot Depicting the Tegner Score of Early ACLR Versus Elective Delayed ACLR After Redefinition

Study or Subgroup	Early ACLR Mean	Early ACLR SD	Early ACLR Total	Delayed ACLR Mean	Delayed ACLR SD	Delayed ACLR Total	Weight	Mean Difference IV, Random, 95% CI
**11.1.1 Follow-up 6 months**								
Mananandhar 2018	4.15	1.45	53	3.72	1.34	54	100.0%	0.43 [-0.10, 0.96]
**Subtotal (95% CI)**			**53**			**54**	**100.0%**	**0.43 [-0.10, 0.96]**
Heterogeneity: Not applicable								
Test for overall effect: Z = 1.59 (P = 0.11)								
**Total (95% CI)**			**53**			**54**	**100.0%**	**0.43 [-0.10, 0.96]**
Heterogeneity: Not applicable								
Test for overall effect: Z = 1.59 (P = 0.11)								
Test for subgroup differences: Not applicable								

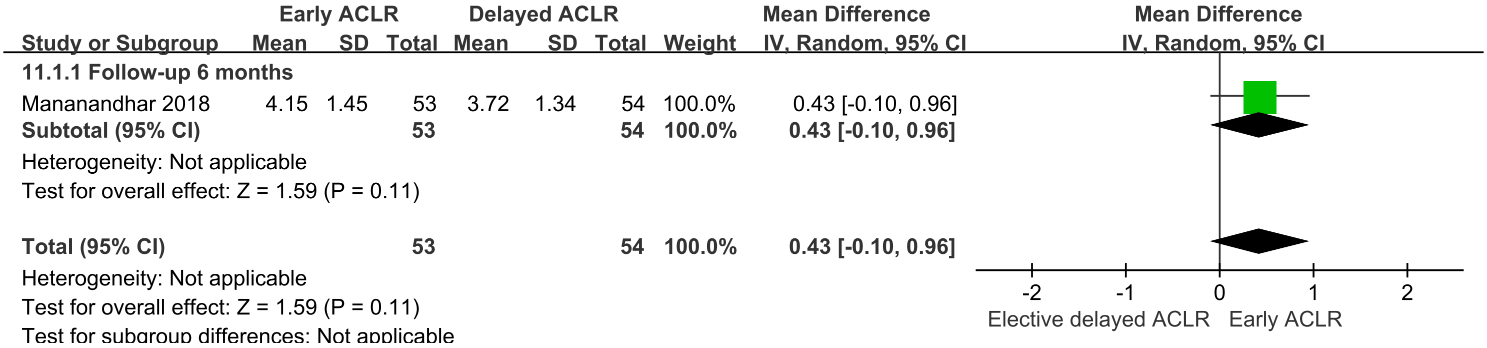

© 2022 Shen X et al. *JAMA Network Open.*

eFigure 12. Forest Plots Depicting the IKDC Score and IKDC Rating Scale of Early ACLR Versus Elective Delayed ACLR After Redefinition

Study or Subgroup	Early ACLR Mean	Early ACLR SD	Early ACLR Total	Delayed ACLR Mean	Delayed ACLR SD	Delayed ACLR Total	Weight	Mean Difference IV, Random, 95% CI
**12.1.1 Follow-up 6 months**								
Mananandhar 2018	69.68	8.14	53	67.14	6.08	51	10.1%	2.54 [-0.21, 5.29]
Reijman 2021	69.6	3.1	85	66.8	3	82	89.9%	2.80 [1.87, 3.73]
**Subtotal (95% CI)**			**138**			**133**	**100.0%**	**2.77 [1.90, 3.65]**
Heterogeneity: Tau² = 0.00; Chi² = 0.03, df = 1 (P = 0.86); I² = 0%								
Test for overall effect: Z = 6.20 (P < 0.00001)								
**12.1.2 Follow-up 1 year**								
Reijman 2021	81.6	1.9	85	74.4	1.9	82	100.0%	7.20 [6.62, 7.78]
**Subtotal (95% CI)**			**85**			**82**	**100.0%**	**7.20 [6.62, 7.78]**
Heterogeneity: Not applicable								
Test for overall effect: Z = 24.48 (P < 0.00001)								
**12.1.3 Follow-up 2 years**								
Reijman 2021	84.7	3	85	79.4	3	82	100.0%	5.30 [4.39, 6.21]
**Subtotal (95% CI)**			**85**			**82**	**100.0%**	**5.30 [4.39, 6.21]**
Heterogeneity: Not applicable								
Test for overall effect: Z = 11.41 (P < 0.00001)								

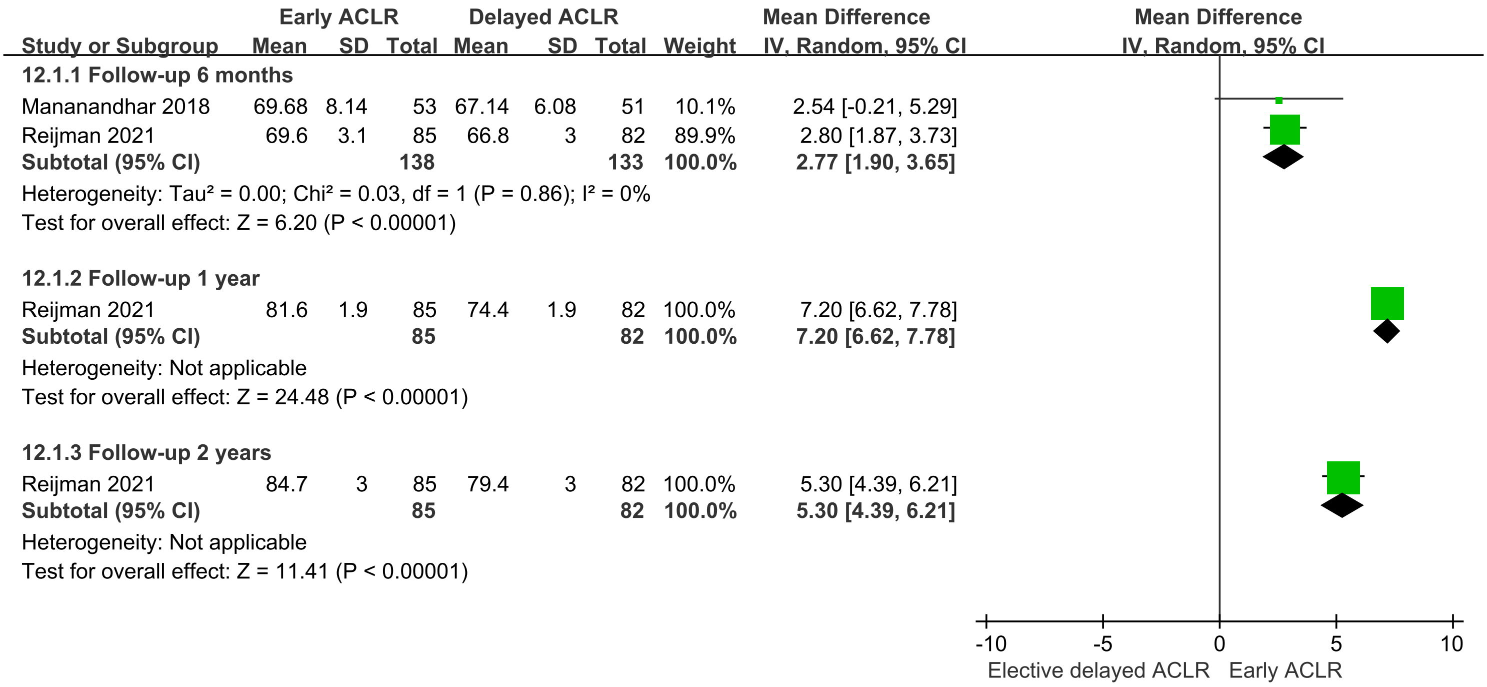

© 2022 Shen X et al. *JAMA Network Open.*

Study or Subgroup	Early ACLR Events	Early ACLR Total	Delayed ACLR Events	Delayed ACLR Total	Weight	Odds Ratio M-H, Random, 95% CI
**12.2.1 Follow-up 6 months**						
Eriksson 2018	27	33	24	34	100.0%	1.88 [0.59, 5.93]
**Subtotal (95% CI)**		**33**		**34**	**100.0%**	**1.88 [0.59, 5.93]**
Total events	27		24			
Heterogeneity: Not applicable						
Test for overall effect: Z = 1.07 (P = 0.28)						
**12.2.2 Follow-up 1 year**						
Meighan 2003	11	13	15	18	29.7%	1.10 [0.16, 7.74]
von Essen (12m) 2020	26	31	27	34	70.3%	1.35 [0.38, 4.79]
**Subtotal (95% CI)**		**44**		**52**	**100.0%**	**1.27 [0.44, 3.67]**
Total events	37		42			
Heterogeneity: Tau² = 0.00; Chi² = 0.03, df = 1 (P = 0.86); I² = 0%						
Test for overall effect: Z = 0.44 (P = 0.66)						
**12.2.3 Follow-up 2 years**						
von Essen (24m) 2020	24	27	28	28	100.0%	0.12 [0.01, 2.50]
**Subtotal (95% CI)**		**27**		**28**	**100.0%**	**0.12 [0.01, 2.50]**
Total events	24		28			
Heterogeneity: Not applicable						
Test for overall effect: Z = 1.36 (P = 0.17)						

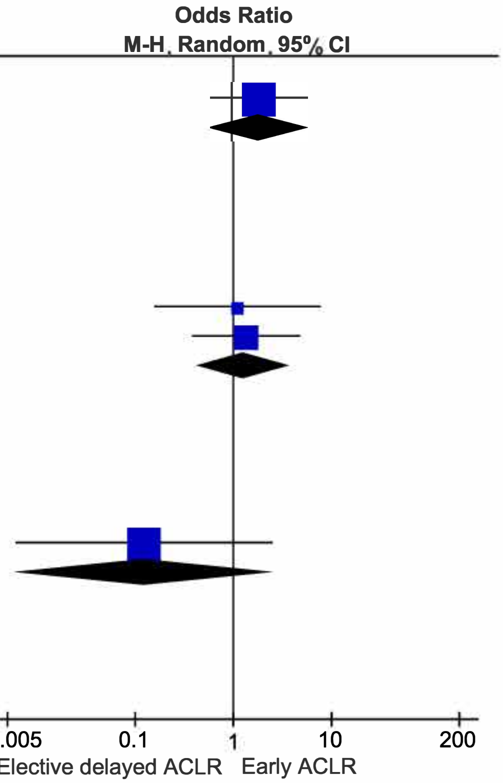

© 2022 Shen X et al. *JAMA Network Open.*

eFigure 13. Forest Plot of the Results of Re-Tear of Early ACLR Versus Elective Delayed ACLR After Redefinition

Study or Subgroup	Early ACLR Events	Early ACLR Total	Delayed ACLR Events	Delayed ACLR Total	Weight	Odds Ratio M-H, Random, 95% CI	Year
Meighan 2003	0	13	1	18	13.6%	0.43 [0.02, 11.47]	2003
Bottoni 2008	1	34	1	35	18.5%	1.03 [0.06, 17.16]	2008
von Essen (12m) 2020	1	34	1	35	18.5%	1.03 [0.06, 17.16]	2020
Reijman 2021	4	85	2	82	49.3%	1.98 [0.35, 11.09]	2021
**Total (95% CI)**		**166**		**170**	**100.0%**	**1.26 [0.38, 4.23]**	
Total events	6		5				

Heterogeneity: Tau² = 0.00; Chi² = 0.71, df = 3 (P = 0.87); I² = 0%
Test for overall effect: Z = 0.38 (P = 0.71)

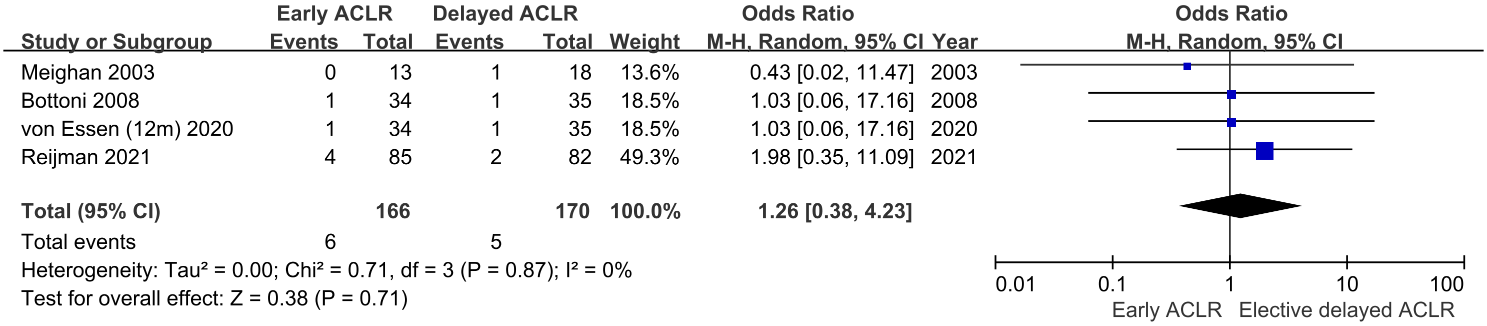

© 2022 Shen X et al. *JAMA Network Open.*

eFigure 14. Forest Plot of the Results Infection of Early ACLR Versus Elective Delayed ACLR After Redefinition

	Early ACLR		Delayed ACLR			Odds Ratio	
Study or Subgroup	Events	Total	Events	Total	Weight	M-H, Random, 95% CI	Year
Meighan 2003	1	13	0	18	32.6%	4.44 [0.17, 118.00]	2003
Bottoni 2008	1	34	0	35	33.6%	3.18 [0.13, 80.79]	2008
Mananandhar 2018	1	53	0	51	33.8%	2.94 [0.12, 73.92]	2018
**Total (95% CI)**		**100**		**104**	**100.0%**	**3.45 [0.53, 22.50]**	
Total events	3		0				

Heterogeneity: Tau² = 0.00; Chi² = 0.03, df = 2 (P = 0.98); I² = 0%
Test for overall effect: Z = 1.30 (P = 0.19)

Odds Ratio
M-H, Random, 95% CI

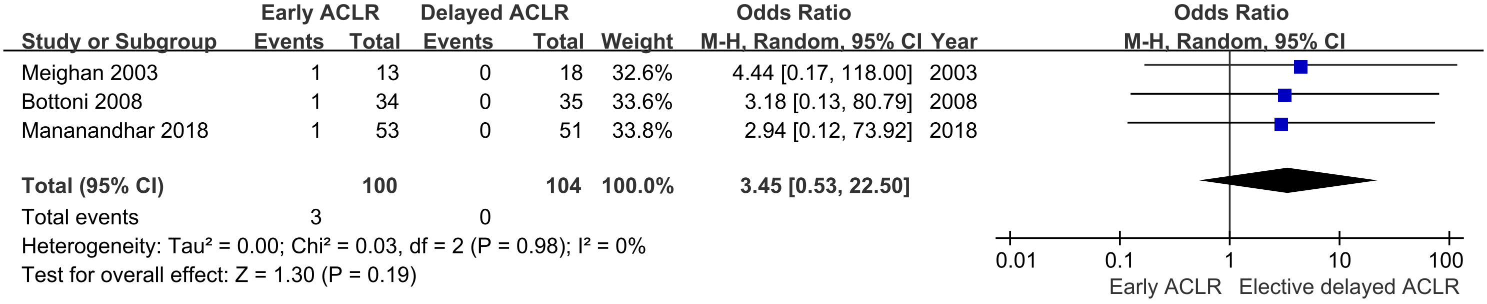

© 2022 Shen X et al. *JAMA Network Open.*
