# Supplementary material for: Optimal Timing of Anterior Cruciate Ligament Reconstruction in Patients With Anterior Cruciate Ligament Tear: A Systematic Review and Meta-analysis
Source: JAMA Netw Open. 2022 Nov 17;5(11):e2242742. doi: 10.1001/jamanetworkopen.2022.42742 (PMC9672975; doi:10.1001/jamanetworkopen.2022.42742)
Supplement: Supplement 2. — Data Sharing Statement [file jamanetwopen-e2242742-s002.pdf]

Data Sharing Statement

Shen. Optimal Timing of Anterior Cruciate Ligament Reconstruction in Patients With Anterior Cruciate Ligament Tear. *JAMA Netw Open*. Published November 17, 2022.
doi:10.1001/jamanetworkopen.2022.42742

Data
Data available: No

Additional Information
Explanation for why data not available: Prof. Qin and Prof. Xiao had full access to all of the data in the study and takes responsibility for the integrity of the data and the accuracy of the data analysis.
